# Protease profiling in fecal samples: a novel non-invasive diagnostic tool for gastrointestinal disorders

**DOI:** 10.1038/s41598-025-32301-6

**Published:** 2025-12-17

**Authors:** Laura Baldassar, Laura Cendron, Sonia Facchin, Carlo Redavid, Brigida Barberio, Alireza Jorkesh, Monica Dettin, Antonella Pasquato, Edoardo Vincenzo Savarino

**Affiliations:** 1 Department of Industrial Engineering, University of Padova, Padova, 35131 Italy; 2 Department of Surgery, Oncology and Gastroenterology, University of Padova, Padova, 35124 Italy; 3 Gastroenterology Unit, Azienda Ospedale Università Padova, Padova, 35124 Italy; 4 Department of Biology, University of Padova, Padova, 35131 Italy

**Keywords:** Fecal proteases, IBD diagnosis, IBS biomarkers, Non-invasive, Gastrointestinal disorders, Biomarkers, Diseases, Gastroenterology, Microbiology

## Abstract

Fecal protease profiling represents a promising frontier in the non-invasive diagnosis of gastrointestinal disorders, particularly inflammatory bowel diseases (IBD), such as Crohn’s disease and ulcerative colitis, and irritable bowel syndrome (IBS). These conditions share overlapping symptoms but differ significantly in etiology and pathology, making accurate differentiation essential for appropriate management. This pilot study investigated protease activity in stool samples using a custom panel of fluorogenic peptide substrates across varying pH conditions to uncover disease-specific enzymatic signatures. Samples from IBD patients revealed broad protease activation involving both serine and cysteine classes, while the small IBS cohort showed a tendency toward a pattern enriched in furin-like serine proteases pattern dominated by furin-like serine proteases, especially at alkaline pH. Notably, one substrate, Ac-RSVL-AMC, showed higher activity in UC than in CD at acidic pH and moderate discriminatory ability in this pilot cohort. Inhibition assays confirmed the enzymatic contribution of furin-like proteases, and follow-up analysis in remission-phase IBD patients indicated persistent dysregulation, suggesting potential biomarker utility beyond active inflammation. The observed substrate-specific activity profiles highlight the importance of sequence context in proteolytic cleavage and underscore the complexity of protease involvement in gastrointestinal pathology. These findings support fecal protease profiling as a promising, rapid and low-cost approach that could contribute to distinguishing IBD from IBS and differentiating IBD subtypes, providing a foundation for future minimally invasive diagnostic strategies that require validation in larger cohorts.

**Supplementary Information:**

The online version contains supplementary material available at 10.1038/s41598-025-32301-6.

## Introduction

Accurate diagnosis is critical for elucidating the underlying mechanisms of symptom elicitation, determining etiology, and predicting complications. It is the foundation for developing targeted therapies and guiding effective treatment strategies, ultimately optimizing patient outcomes and reducing unnecessary interventions. Given these premises, the vast diversity of gastrointestinal diseases poses significant diagnostic challenges, as many conditions exhibit overlapping symptoms. This complexity often hampers accurate categorization and differentiation, complicating treatment strategies and underscoring the need for more precise diagnostic tools.

Among the most prevalent gastrointestinal disorders are ulcerative colitis (UC) and Crohn’s disease (CD), collectively termed inflammatory bowel disease (IBD), along with irritable bowel syndrome (IBS). IBD affects over 10 million individuals worldwide^1–3^, while IBS is estimated to impact 10–23% of the global population^4^, highlighting the substantial healthcare burden associated with these conditions. IBD is driven by a complex interplay of genetic^5–8^, environmental^9^, and immune factors^10–13^. Dysbiosis in the gut microbiota also contributes to disease onset and progression^14–21^. Clinically, CD and UC share symptoms like abdominal pain but differ in pathology. CD affects any part of the gastrointestinal tract with transmural, patchy inflammation, while UC is confined to continuous inflammation of the colon and rectum. These distinctions complicate diagnosis and treatment.

IBS is a prevalent functional gastrointestinal disorder, characterized by recurrent episodes of abdominal pain, bloating, and altered bowel habits^4^. Unlike IBD, IBS does not involve chronic inflammation or structural damage, yet its pathophysiology remains complex and multifactorial. Emerging evidence suggests that visceral hypersensitivity, disruptions in the gut-brain axis, altered microbiota, and psychological factors such as stress and anxiety contribute to symptom onset and severity^17,22–24^. Additionally, immune dysregulation and genetic predispositions may play a role in some patients^11^. IBS is categorized into subtypes based on predominant symptoms: diarrhea-predominant (IBS-D), constipation-predominant (IBS-C), and mixed type (IBS-M), each impacting quality of life and imposing a substantial socioeconomic burden. Despite extensive research, treatment remains symptomatic, focusing on dietary interventions, pharmacotherapy, and psychological therapies, reflecting the challenge of addressing its complex etiology.

Diagnostic tools for IBD and IBS range from invasive procedures such as biopsies and colonoscopies to non-invasive tests, including (C-reactive protein) (CRP) and fecal calprotectin However, these non-invasive markers lack specificity, making it difficult to accurately distinguish between gastrointestinal disorders and enhance patient management^25,26^.

Proteases, enzymes responsible for catalyzing the cleavage of protein substrates, are categorized into serine, cysteine, aspartic, glutamic, threonine, and metalloproteases based on their catalytic mechanisms. In the gastrointestinal tract, proteases are indispensable for processes such as protein digestion, immune modulation, and the maintenance of gut homeostasis. However, in the context of gastrointestinal diseases, their dysregulation is well-documented, leading to compromised mucosal barrier integrity, disruption of epithelial tight junctions, and the amplification of inflammation (Table S1). Elevated expression of serine proteases, including neutrophil elastase, proteinase 3, cathepsin G, tryptase, and chymase, has been observed in mucosal biopsies from patients with IBD, with high elastase activity detected even in non-inflamed areas^27^, highlighting their potential as therapeutic targets^28,29^. Furthermore, heightened fecal protease activity (PA), particularly driven by bacterial elastase, has been reported in UC. Notably, elevated fecal PA correlates with disease severity and may precede clinical diagnosis, positioning it as a promising non-invasive biomarker for early disease detection^30^. In IBS, increased levels of trypsin and tryptase are known to heighten visceral sensitivity by activating Protease-Activated Receptor 2 (PAR-2), which amplifies pain signals and contributes to the discomfort experienced by IBS patients^31^. Interestingly, in diarrhoea-predominant irritable bowel syndrome (IBS-D), elevated serine protease activity, particularly from trypsin and chymotrypsin, disrupts the gut protective barrier and worsens visceral sensitivity through PAR-2 activation. Blocking these proteases in experimental models has been shown to prevent these effects, making them potential therapeutic targets for IBS-D^32^. Fecal samples from IBS-D patients show increased trypsin-like activity, which cleaves lysine and arginine, along with reduced elastase-like activity, which normally targets serine and glycine. These changes in protease activity are closely linked to gut barrier dysfunction and increased sensitivity, suggesting their use as potential biomarkers for diagnosing IBS-D. Additionally, machine-learning models based on fecal protease activity have demonstrated high accuracy in distinguishing IBS-D patients from healthy individuals, highlighting their promise in advancing diagnostic approaches for IBS^33^. While dysregulated intestinal serine protease activity is primarily linked to IBS, a broad spectrum of proteases, including serine, cysteine, and metalloproteinases, is involved in IBD. However, there is no clear evidence identifying a specific protease class uniquely dysregulated in CD or UC, complicating the understanding of their contributions to disease progression. Furthermore, in both IBD and IBS, the interplay between host and microbiome-derived proteases likely exacerbates disease pathology, underscoring the need to better understand their roles in gastrointestinal disorders.

In this pilot study, we evaluated the enzymatic activity of proteases in stool samples from 12 patients with CD, 14 with UC, 4 with IBS, and 12 healthy controls, using 14 different fluorogenic peptide substrates. Our aim was to identify distinct patterns of proteolytic activity that could form the basis of a rapid, cost-effective diagnostic tool for distinguishing these conditions.

## Results

### Fluorogenic substrates assays uncover unique protease activity profiles in IBD

**Fig. 1:**
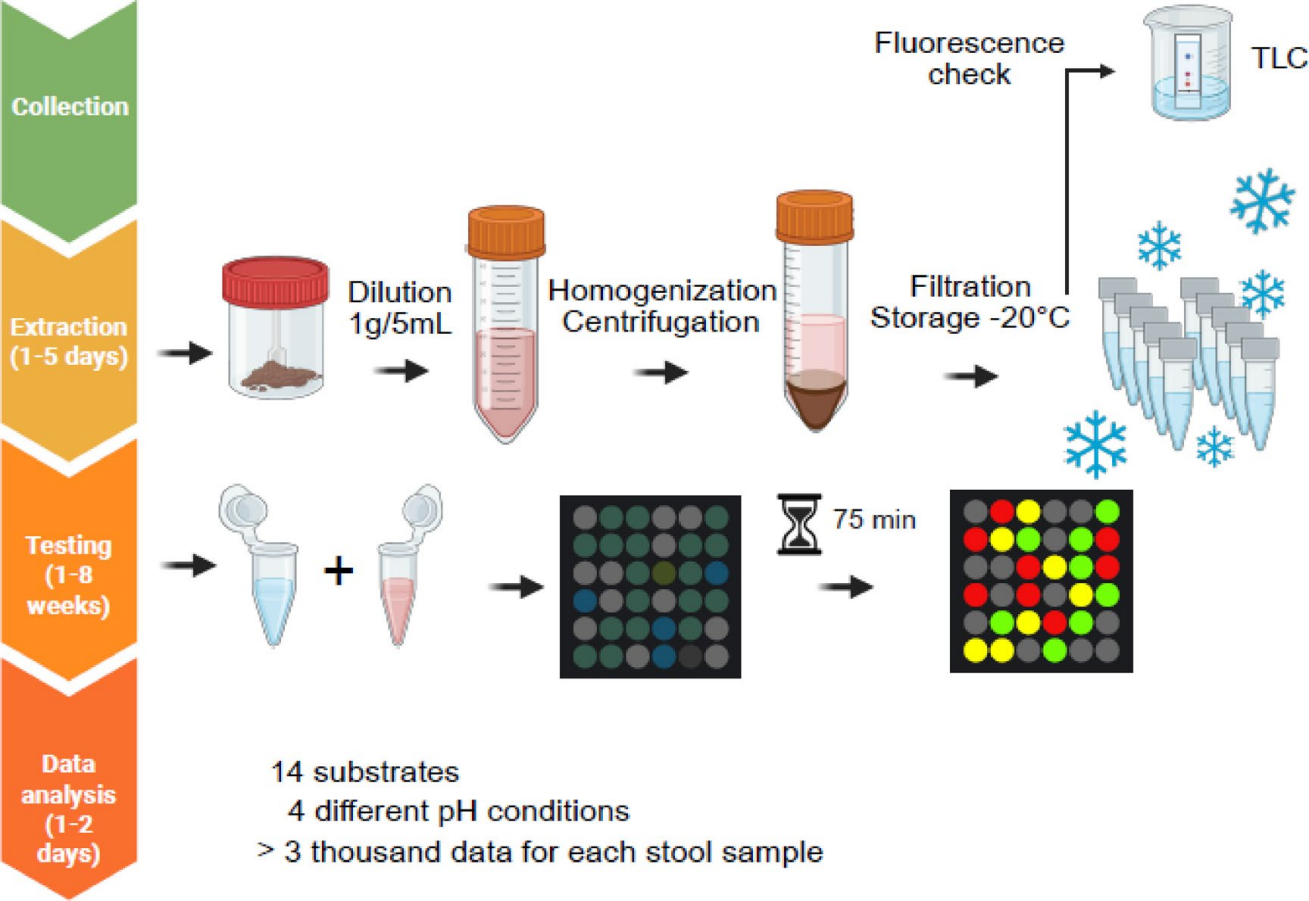
Experimental workflow for protease activity analysis in stool samples. Stool samples were collected from patients with inflammatory bowel disease (IBD), irritable bowel syndrome (IBS), and healthy controls (HC). Samples underwent extraction, dilution (1 g/5 mL), homogenization, centrifugation, and filtration before storage at − 20 °C. Fluorogenic peptide substrates (5 µM) were incubated with stool extracts (1:20 v/v dilution) under four pH conditions (5.5, 6.5, 7.5, 8.0), and fluorescence was measured every 5 min for 75 min using a plate reader (λ_ex_ = 365 nm, λ_em_ = 465 nm). Data were normalized, and enzymatic activity was calculated as the slope of the linear phase of fluorescence increase (RFU/min). Statistical analyses were performed using GraphPad Prism with a 95% confidence interval.

**Fig. 2:**
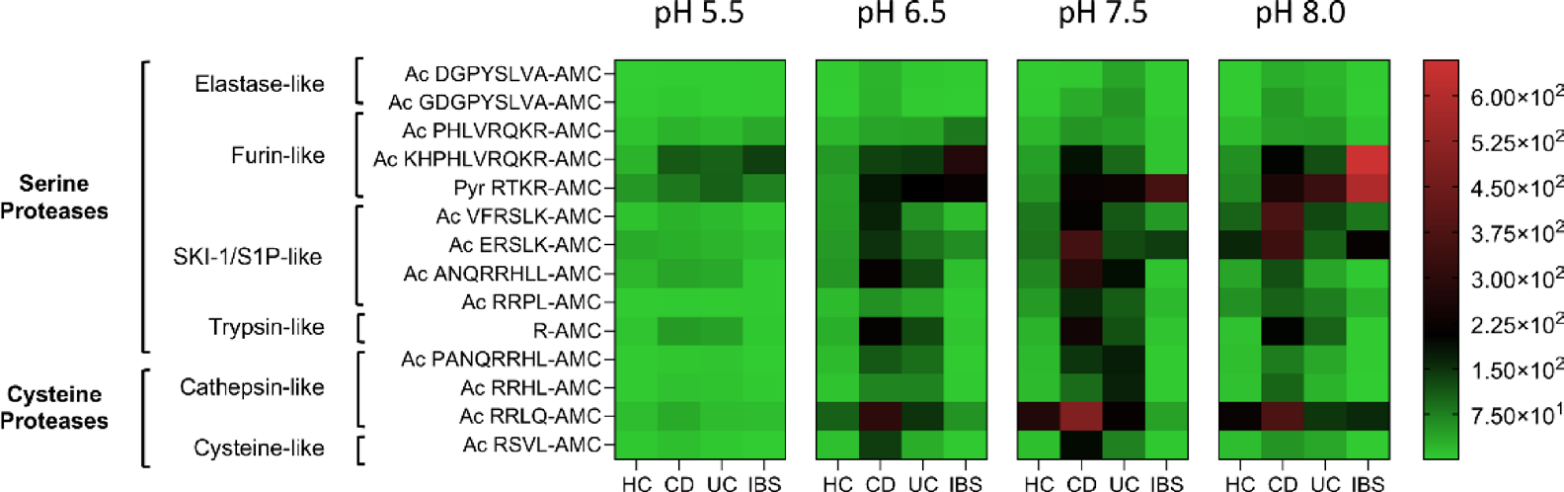
Heatmap of serine and cysteine protease activity across patient groups. Protease activity was assessed in stool samples from Healthy Controls (HC), Crohn’s Disease (CD), Ulcerative Colitis (UC), and Irritable Bowel Syndrome (IBS) patients using 14 fluorogenic peptide substrates at pH 5.5, 6.5, 7.5, and 8.0. Each row corresponds to a specific peptide substrate, and each column represents the mean protease activity value for all samples within a given cohort, averaged from the individual measurements shown in Figure S4. Color intensity indicates relative protease activity (RFU min⁻¹) on a 0–600 RFU min⁻¹ scale, with warmer tones reflecting higher activity. Serine protease substrates (elastase-like and furin-like) are displayed at the top, and cysteine protease substrates (cathepsin-like) at the bottom. This mean-based representation highlights group-level enzymatic trends rather than individual variability. While IBD samples (UC and CD) exhibited distinct activity profiles—with UC showing relatively higher cathepsin-like activity—the smaller IBS cohort (*n* = 4) showed increased cleavage of furin-like substrates, although this trend should be interpreted cautiously given the limited sample size. Data were normalized for cross-comparison (see Fig. S4 for individual values).

Clinical metadata for individual UC and CD patients, including disease phenotype and activity markers, are reported in Table S2. To investigate protease activity in stool extracts from IBD (UC and CD) and IBS patients (serving as a non-inflammatory control cohort), we utilized a custom-designed panel of 14 fluorogenic peptide substrates (Table 1). These sequences were derived from proteins critical to gastrointestinal integrity and inflammation, such as Contactin-1, Desmoglein-2, and ATF6, chosen for their relevance in disease mechanisms and accessibility for enzymatic cleavage. Each peptide substrate was incubated with 20 µL of filtered stool extracts prepared from 1 g of fecal matter homogenized in 5 mL of MilliQ water. The reactions were conducted under varying pH conditions (5.5, 6.5, 7.5, and 8.0) to maximize the capture of a wide range of protease activities. Fluorescence was monitored over time, with the intensity directly proportional to the active protease levels in the stool extract (Fig. 1). Intrinsic fluorescence was evaluated for each stool extract prior to substrate addition. In some samples, a strong substrate-independent signal was observed, which persisted after additional clarification by high-speed centrifugation, indicating the presence of soluble fluorescent compounds rather than particulate scattering. Samples showing excessively high intrinsic fluorescence were excluded from downstream analyses (Fig. S1). About 20% of samples showed background fluorescence, likely from incomplete mesalazine absorption (Fig. S1, and S2), and were corrected for accuracy. Fluorescent data were summarized in the heatmap shown in Fig. 2, which presents the mean proteolytic activity profiles across stool samples from different patient cohorts – Healthy Controls (HCs), UC, CD, and IBS. Each row corresponds to a specific peptide substrate, and each column represents the average activity value for all samples within a given cohort under the indicated pH conditions. The color intensity indicates relative protease activity (RFU min⁻¹), with warmer colors representing higher activity levels.

**Table 1:** Overview of custom-designed peptide substrates used in the study.

		Sequence	Custom	Derivation	pH optimum
Serine protease	Elastase-like Substrates	DGPYSLVA-AMC	Yes	CNTN1 (791–799)	7.5–8.5
		GDGPYSLVA-AMC	Yes	CNTN1 (790–799)	
	Furin-like Substrates	PHLVRQKR-AMC	Yes	DSG2 (42–49)	6.5-8.0
		KHPHLVRQKR-AMC	Yes	DSG2 (40–49)	
		RTKR-AMC	ca.	-	
	SKI-1/S1P-like Substrates	RRPL-AMC	Yes	Multiple proteins	7.5-8.0
		VFRSLK-AMC	Yes	PLRG1 (14–19) SKI-1/S1P (142–147)	
		ANQRRHLL-AMC	Yes	ATF6 (412–419)	
		ERSLK-AMC	Yes	GIIβ (381–385) MGAM (1409–1413)	
	Trypsin-like substrate	R-AMC	ca.	-	
Cysteine protease	Cathepsin-like Substrates	RRHL-AMC	Yes	Multiple proteins	5.0–7.0
		RRLQ-AMC	Yes	Multiple proteins	
		RSVL-AMC	Yes	Multiple proteins	
		PANQRRHL-AMC	Yes	ATF6 (411–418)	

Each substrate is labeled with 7-amido-4-methylcoumarin (AMC) at the C-terminus, allowing for the fluorescence-based detection of protease activity upon cleavage. The table categorizes substrates based on their protease specificity, including serine proteases (elastase-like, furin-like, and SKI-1/S1P-like) and cysteine proteases (cathepsin-like). Additionally, the table provides information on the optimal pH range for each substrate and indicates whether the substrate is custom-made or commercially available; ca., commercially available, Contactin-1, CNTN1; Desmoglein-2, DSG2; Glucosidase II beta subunit, GIIβ; Maltase-glucoamylase isoform 2, MGAM; PLRG1, pleiotropic regulator 1; Subtilisin-kexin-isozyme-1 (SKI-1) / Site-1 protease (S1P); Activating transcription factor 6, ATF6.

The heatmap in Fig. 2 summarizes mean protease activity values for each cohort, calculated from the individual measurements shown in Figure S4, and is intended to illustrate overall enzymatic trends rather than inter-individual variability. While individual patient measurements displayed considerable variability (as illustrated in Fig. S4), this heterogeneity is consistent with previous reports describing inter-individual differences in fecal protease activity among IBS patients^34^. The use of mean values thus highlights dominant enzymatic trends characteristic of each cohort, while detailed variability is provided in the supplementary figures.

The protease activity profiles observed across patient samples revealed differences between HC, CD, UC, and IBS. Given the small sample size of the IBS group, comparisons involving this cohort were interpreted cautiously and used primarily for descriptive purposes. Healthy controls exhibited negligible protease activity across all substrates, consistent with the absence of inflammation or tissue damage, establishing a robust baseline for comparison (Fig. 2, Fig. S4). By contrast, samples from CD and UC patients showed markedly elevated protease activity, each displaying unique enzymatic patterns, …whereas IBS samples, though also exhibiting increased overall activity, tended to display a profile that differed from both healthy individuals and IBD patients.

At pH 5.5, protease activity was the lowest across all conditions, yet substrates such as Ac-KHPHLVRQR-AMC and Pyr-RTKR-AMC remained active, albeit with reduced fluorescence signals. Notably, Ac-RSVL-AMC was the only substrate capable of distinguishing between UC and CD samples at this pH, suggesting its potential as a biomarker for differentiating these two IBD subtypes Ac-RSVL-AMC showed higher mean activity in UC than in CD. In this exploratory cohort, ROC analysis yielded an AUC of 0.73 for differentiating UC from CD (Fig. S5), indicating moderate discriminatory potential that warrants evaluation in larger, independent cohorts (Fig. 2, Fig. S4).

As the pH increased to 6.5, protease activity rose across most substrates, in line with the pH profiles expected for many proteolytic enzymes. Substrates such as Ac-KHPHLVRQR-AMC and Pyr-RTKR-AMC demonstrated significant cleavability in both IBD and IBS samples relative to healthy controls, supporting the hypothesis that furin-like serine proteases play a key role in these conditions. CD and, to a lesser extent, UC samples also exhibited enhanced cleavage of substrates including Ac-VFRSLK-AMC, Ac-ANQRRHLL-AMC, Ac-RRLQ-AMC, and R-AMC (Fig. 2, Fig. S4).

At pH 7.5, the activity profile remained consistent, with CD and UC samples exhibiting significant protease activity, particularly for substrates mentioned above. Interestingly, UC samples exhibited a marked preference for Ac-PANQRRHL-AMC and Ac-RRHL-AMC at this pH, which was unique to this condition and not observed at lower pH levels (Fig. 2, Fig. S4).

At pH 8.0, IBS samples showed their highest furin-like protease activity, particularly for Pyr-RTKR-AMC and Ac-KHPHLVRQR-AMC, often exceeding the activity observed in both HC and IBD samples in this cohort. Although variability increased within the CD and UC groups and the IBS sample size was limited, these trends suggest that furin-like activity may contribute to distinguishing IBS from IBD, a hypothesis that requires confirmation in larger studies. IBD samples, meanwhile, demonstrated a more restricted range of substrate cleavage at this pH, retaining activity primarily against furin-like substrates (Ac-RRLL-AMC), along with Ac-R-AMC, Ac-VFRSLK-AMC, and Ac-ERSLK-AMC (Fig. 2, Fig. S4).

In our IBS samples, trypsin-like activity was detectable but comparatively low, whereas furin-like substrates showed markedly higher cleavage. This pattern does not contradict previous studies reporting elevated trypsin activity in IBS-D, as most of those investigations focused on a narrow set of serine protease substrates and did not assess furin-like activity in parallel. By using a broader panel of substrates, our approach may therefore reveal a relative predominance of furin-like activity that was not captured by earlier assays. Nevertheless, given the small cohort size and lack of subtype stratification, these observations should be considered preliminary and require confirmation in larger IBS populations.

Of note, the pH dependence of Ac-KHPHLVRQKR-AMC cleavage did not follow a strictly monotonic pattern in IBS. Activity for this furin-like substrate was clearly increased at pH 6.5 and pH 8.0, while at pH 7.5 the values overlapped more with those observed in UC and CD. Given that stool extracts contain a mixture of host- and microbiota-derived proteases and endogenous inhibitors, and that basic residue–rich substrates can be recognized by several proprotein convertases and trypsin-like enzymes, this non-linear profile likely reflects the composite contribution of multiple proteases with partially distinct pH optima rather than the presence of two separate furin-like populations. Importantly, when considering the entire panel of furin-like substrates and pH conditions, IBS samples tended to display a coherent serine protease–dominated signature distinct from IBD and healthy controls.

**Fig. 3:**
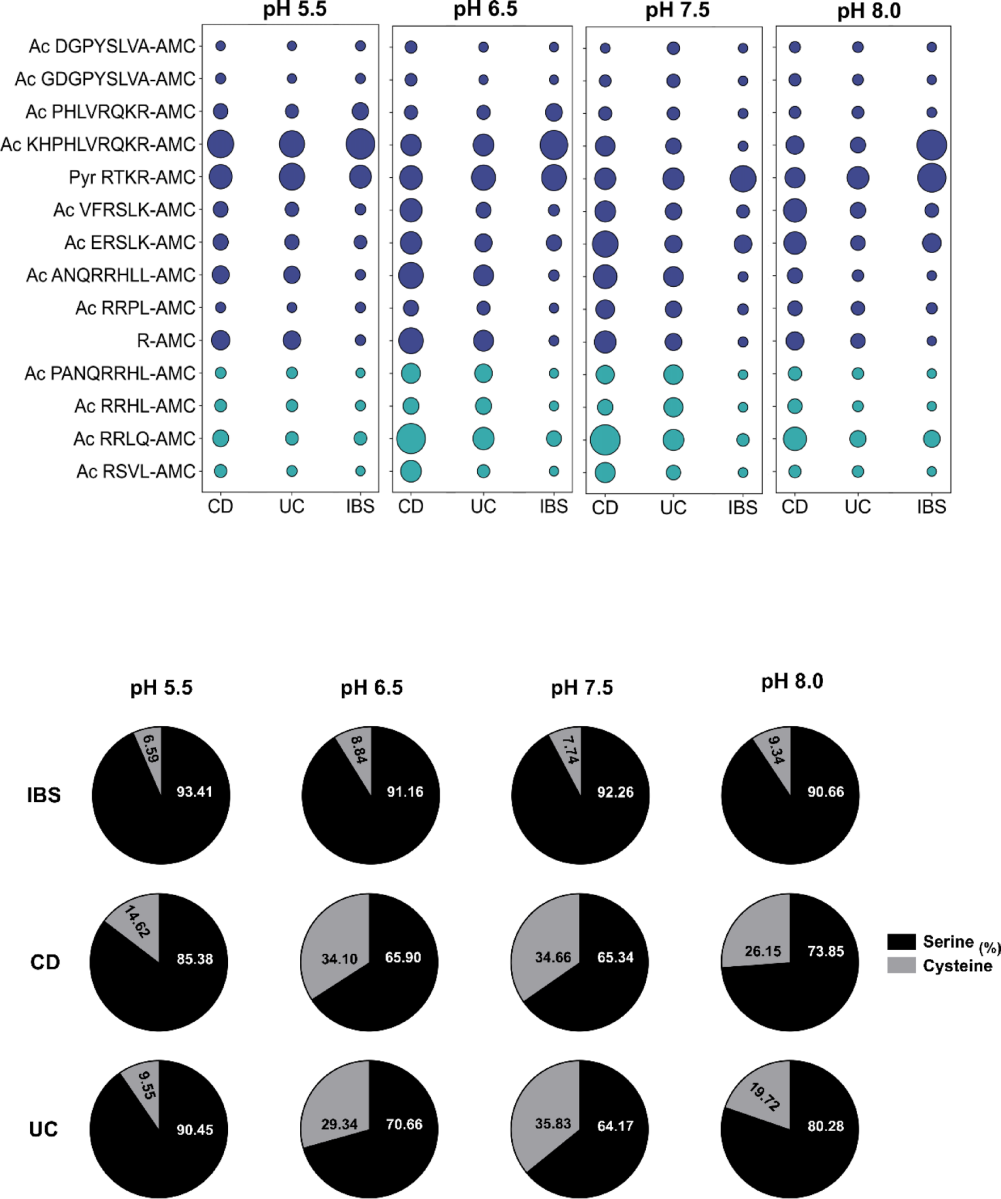
Normalized enzymatic activity profiles by substrate and protease class. Enzymatic activity data were normalized and analyzed by individual substrates (**a**) and by grouping them into serine or cysteine protease classes (**b**) across different pH conditions. Distinct protease activity profiles were observed among HC, CD, UC, and IBS groups, with notable differences depending on substrate type and pH.

The enzymatic profiles were further analyzed by clustering the contributions of cysteine and serine protease-specific substrates, both by class (Fig. 3a) and individually (Fig. 3b), normalized across pH conditions. Results revealed that IBD samples (UC and CD) exhibited a balanced enzymatic profile with cysteine proteases making substantial contributions. At pH 6.5 and 7.5, cysteine protease activity exceeded 30% in IBD cohorts, reflecting the involvement of multiple protease classes in inflammatory processes (Fig. 3a). These findings were driven by substrates such as Pyr-RTKR-AMC, Ac-VFRSLK-AMC, Ac-ERSLK-AMC, Ac-ANQRRHLL-AMC, Ac-RRLQ-AMC, R-AMC, and Ac-KHPHLVRQKR-AMC (Fig. 3b). Notably, Ac-PHLVRQKR-AMC, which is only two amino acids shorter than Ac-KHPHLVRQKR-AMC, was a poor substrate under all conditions tested, underscoring the critical influence of peptide length and sequence on substrate efficacy. Interestingly, IBS samples, used as non-inflammatory controls, exhibited a protease signature predominantly driven by serine proteases, particularly furin-like enzymes, that tended to differ from IBD profiles across pH conditions in this cohort. This apparent divergence suggests that UC and CD possess proteolytic landscapes that are not identical to IBS, despite IBS also displaying elevated protease activity compared to healthy controls, albeit in a different pattern from UC and CD.

In summary, these findings highlight the distinct proteolytic landscapes of UC and CD compared to IBS and healthy controls, underscoring the potential of specific protease activity profiles as biomarkers for differentiating gastrointestinal disorders. However, further experiments with larger patient cohorts (including diagnostically challenging UC/CD cases) and an expanded panel of peptide substrates are needed to extend these observations and enhance diagnostic precision.

### Furin-like protease activity as a defining feature of IBS

IBS-related protease activity, often linked to serine proteases, is well-documented. Using the furin-specific substrate Pyr-RTKR-AMC, we confirmed furin-like activity through inhibition with chloromethyl ketone (CMK), a selective furin inhibitor. The protease activity of IBS stool, possibly attributed to serine proteases, is well-documented in the literature.

**Fig. 4:**
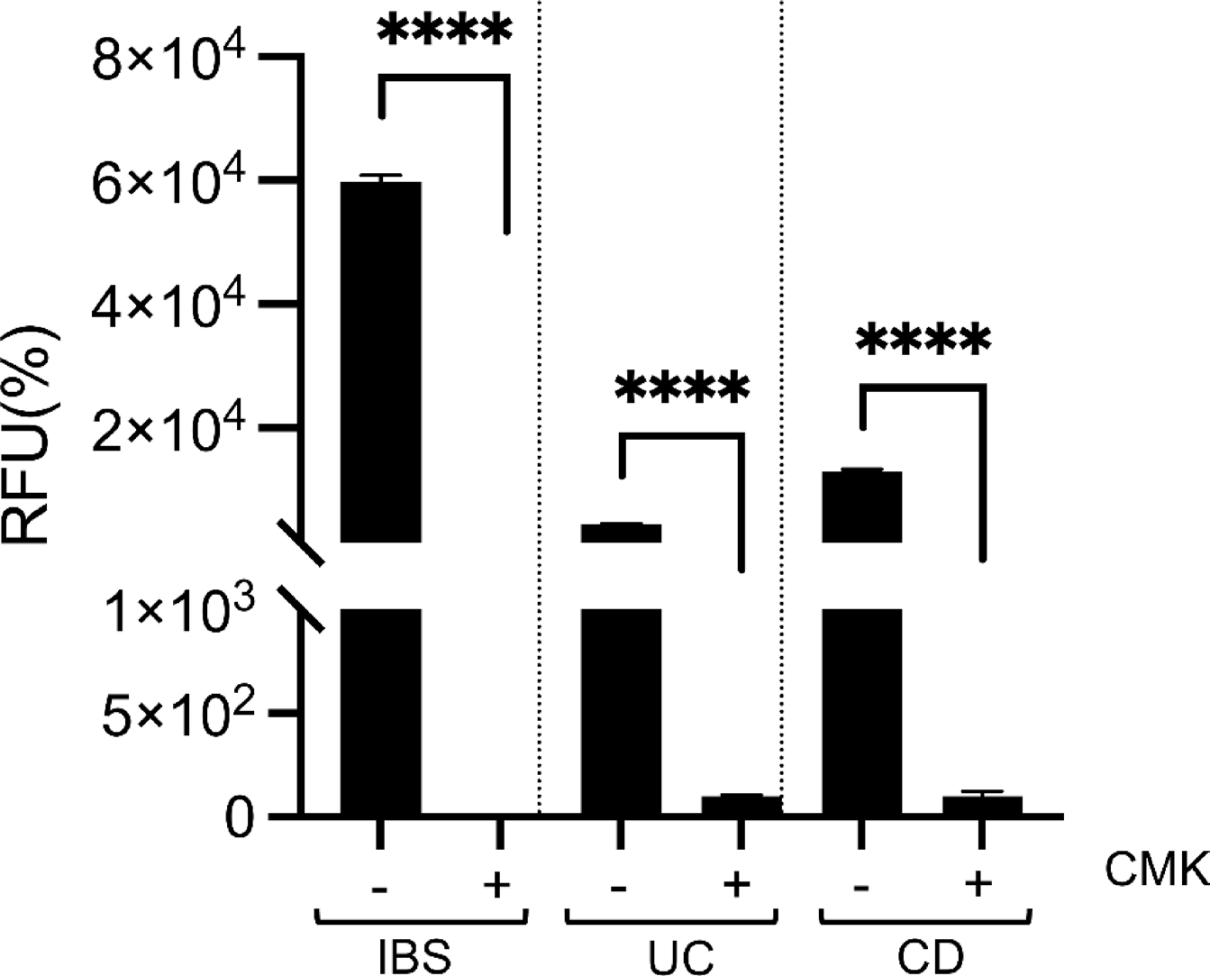
Inhibition of furin-like protease activity in IBS, UC, and CD stool samples. Protease activity was assessed using the substrate Pyr-RTKR-AMC (5 µM) at pH 7.5, with or without the furin inhibitor chloromethyl ketone (CMK, 2 µM). Fluorescence was measured over 75 min, and enzymatic activity was calculated as RFU/min. Data are expressed as mean ± SD and normalized to control reactions. CMK significantly inhibited furin-like activity, with statistical comparisons performed using an unpaired t-test (****p < 0.0001).

Stool samples from IBS, CD, and UC patients were tested in triplicate with 2 µM CMK at pH 7.5, using Pyr-RTKR-AMC as the substrate. MK markedly reduced activity in all groups, confirming a significant role for furin-like proteases in both IBD and IBS (Fig. 4).

Further studies with larger sample sizes, particularly for IBS, are needed to confirm these findings and better characterize the role of furin-like proteases in gastrointestinal disorders.

### Elevated protease activity can be detected in both acute and remissive phases of the disease

To determine whether protease upregulation is specific to the acute phase or a consistent feature of IBD, we performed follow-up tests on three patients (two with CD, one with UC) previously sampled during an acute episode and later during remission. The same substrates and pH conditions were used, and paired t-tests compared activity levels between phases.

**Fig. 5:**
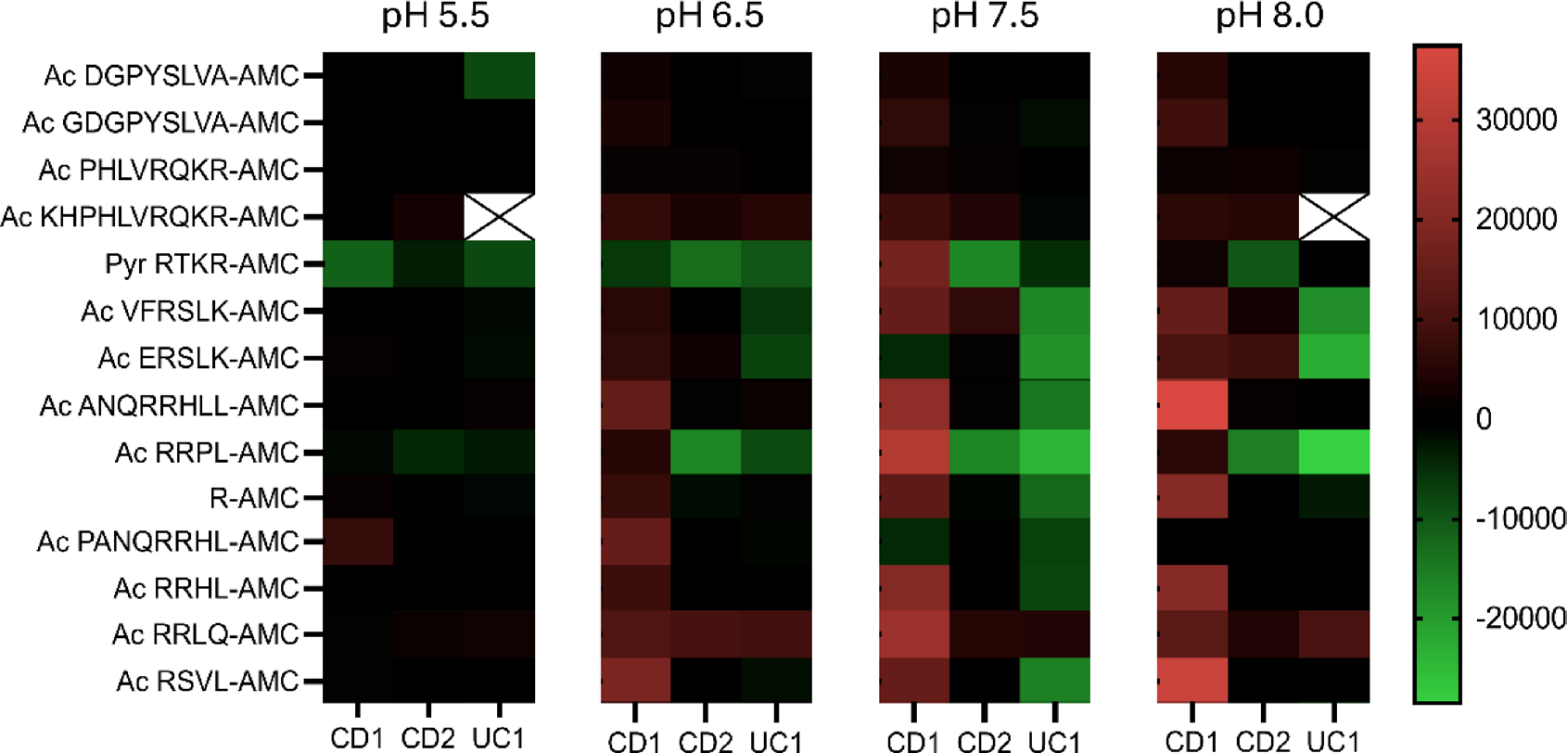
Enzymatic activity in follow-up (F-U) stool samples from IBD patients in remission. Protease activity was analyzed in follow-up samples collected during remission and compared to activity levels measured during the acute phase. Fourteen fluorogenic substrates were tested at pH 5.5, with fluorescence recorded every 5 min for 75 min. Enzymatic activity (RFU/min) was calculated and normalized for cross-comparison. Differences in activity between acute and remission phases were statistically analyzed using an unpaired t-test.

Figure 5 shows protease activity at pH 5.5, 6.5, 7.5, and 8.0. One CD patient showed increased activity in remission, the other a decrease; the UC patient also showed reduced activity. Despite these individual differences, all samples maintained higher activity than healthy controls, suggesting persistent dysregulation regardless of disease phase.

These data offer crucial preliminary insights, suggesting that protease activity does not exhibit a consistent correlation with disease phase (acute vs. remission). Notably, the trends in protease activity—whether increased or decreased—were consistent across all substrates, implying that the total protease load fluctuated rather than specific classes of proteases being differentially regulated between phases.

## Discussion

This study explores a novel, non-invasive approach based on fecal protease activity to differentiate between ulcerative colitis, Crohn’s disease, and irritable bowel syndrome. By profiling enzymatic activity across a panel of fluorogenic peptide substrates at different pH levels, we identified trends for each condition. This approach offers a promising complement to invasive diagnostic procedures like colonoscopy^25^, although its potential clinical role will need to be defined in larger, prospective studies.

Protease dysregulation is a well-established feature of gastrointestinal disorders, with IBD displaying distinct proteolytic activity compared to IBS and healthy controls^31,35^. Our findings provide new insight into how proteases may contribute to IBD pathology. While previous studies have reported elevated serine protease activity as a hallmark of IBS, our data should be interpreted with caution given the limited size of the IBS cohort. Within this constraint, IBS samples showed a tendency toward increased cleavage of certain serine substrates, whereas IBD samples exhibited a broader and more balanced protease profile, involving both serine and non-serine enzymes across multiple substrates and pH conditions. This pattern reflects the multifactorial nature of inflammation in UC and CD^35^.

We also observed substantial variability in protease activity between individual IBD samples, consistent with previous findings by Jablaoui et al. (2020)^35^. In that study, fecal serine protease activities—including trypsin-, elastase-, proteinase 3-, and cathepsin G-like—were significantly increased in both ulcerative colitis and Crohn’s disease compared to healthy controls, but with broad inter-patient ranges that mirrored the heterogeneous inflammatory status of these disorders. Such heterogeneity likely reflects differences in mucosal inflammation, microbial composition, and disease phase (active vs. remission), and aligns with the variability observed in our cohorts. Comparable inter-individual variability has also been reported in IBS patients^34^, where both host and microbial proteases contribute to distinct yet overlapping activity profiles.

The recent work by Soussou et al. (2023)^34^ provides important context for interpreting our IBS findings. In a larger Tunisian IBS cohort, they reported significant increases in serine proteases—including trypsin-like, elastase-like, and cathepsin G–like activities—as well as elevated metalloproteases. Their results reinforce the concept of pronounced serine protease dysregulation in IBS. While our study detected measurable trypsin-like activity, IBS samples in our cohort showed relatively stronger cleavage of furin-motif substrates when assessed alongside a broader substrate panel. This difference likely reflects methodological distinctions: Soussou et al. focused primarily on serine-protease–targeted substrates, whereas our multi-substrate, multi-pH approach captures a wider range of protease specificities, allowing overlapping enzymatic activities to emerge in parallel. Given our limited IBS sample size, these observations should be considered preliminary but complementary to the established literature.

A central methodological aspect of our study is that protease activity is inferred from the cleavability of specific peptide motifs, rather than from the assumption that each substrate is cleaved by a single enzyme. In stool samples, enzymes with overlapping specificities may contribute to cleavage of the same motif. For this reason, we deliberately employed a broad panel of substrates covering distinct sequence classes, enabling us to capture the composite protease landscape across IBD, IBS, and healthy controls. This multi-substrate, multi-pH approach is essential to disentangle the relative contributions of protease families and to avoid the oversimplification inherent in single-enzyme assays.

This conceptual framework, together with future expansion of substrate panels and larger, well-characterized patient cohorts, may help uncover novel disease mechanisms and guide the development of more reliable diagnostic tools.

Importantly, IBD-associated proteases demonstrated selective substrate recognition. For example, the DSG2-derived substrate was robustly cleaved, while the contactin-1 CNTN-1-derived substrate showed minimal activity. Even small sequence differences significantly influenced cleavage efficiency: Ac-KHPHLVRQKR-AMC was cleaved, whereas its shorter version, Ac-PHLVRQKR-AMC, was not—despite differing by only two amino acids. This emphasizes the importance of sequence context in substrate specificity^36^. These results argue against indiscriminate enzymatic activity and instead point to targeted, disease-relevant protease functions. This reflects the altered biochemical landscape characteristic of IBD, where multiple protease classes are dysregulated (e.g^35^. , .

Furin-like proteases were identified as key contributors to the observed protease activity through specific inhibition assays. This is noteworthy, as furin has primarily been studied in gastrointestinal cancers^37^, and its role in IBD and IBS remains underexplored. Furin plays a regulatory role in immune responses by activating pro-cytokines and growth factors such as TGF-β and TNF-α, facilitating their function in inflammatory signaling. It also influences tissue remodeling by modulating matrix metalloproteinases^38^. Our detection of furin-like activity in IBD aligns with previous reports linking furin dysregulation to inflammatory conditions. In IBS, used here as a positive control, we also observed distinct furin-like activity. As a serine protease, furin fits well within the established protease profile of IBS^32^, consistent with of our findings. Although the number of IBS samples was limited, the upregulation of furin-like activity in this group is consistent with known features of IBS, such as increased intestinal permeability and altered gut-brain axis signaling^4^. Furin’s ability to activate neuropeptides may contribute to visceral hypersensitivity, a core symptom of IBS^39^, and echoes its emerging role in neurodegenerative and neuropsychiatric conditions^40^. The observed furin activity in both IBD and IBS suggests potential diagnostic and therapeutic implications. Recent evidence supporting the efficacy of serine protease inhibitors in IBS symptom relief^41^ further underscores this point. Targeted modulation of furin or related enzymes could offer more precise treatment strategies with fewer side effects, suggesting potential translational interest of our findings.

Protease activity profiles also revealed differences between CD and UC. At pH 5.5, the substrate Ac-RSVL-AMC showed higher mean activity in UC than in CD, suggesting a potential group-level distinction. In this pilot cohort, a ROC analysis yielded an AUC of 0.73 (Supplementary Fig. S5), indicating moderate discriminatory ability. Given the limited sample size and the inherent biological variability of fecal protease activity, such moderate AUC values are expected, and these results should be interpreted as preliminary and hypothesis-generating rather than as evidence of standalone diagnostic performance. UC samples tended to exhibit higher overall protease activity than CD, consistent with more pronounced mucosal dysregulation, but substantial inter-patient variability in both groups underscores the need for longitudinal sampling. A single time-point is unlikely to capture the dynamic and episodic nature of protease activity in IBD, whereas repeated measurements may better reflect fluctuations related to disease progression or remission.

In this context of biological heterogeneity, the behaviour of individual substrates must be interpreted cautiously. For example, the non-linear pH profile of Ac-KHPHLVRQKR-AMC in IBS samples—showing increased activity at pH 6.5 and 8.0 but not at 7.5—illustrates that no single substrate–pH combination is likely sufficient on its own for robust disease classification. Accordingly, any screening or diagnostic strategy based on fecal protease activity will require a composite approach, integrating signals from multiple substrates and at least two pH conditions to buffer local fluctuations and inter-individual variability. Within such a multidimensional profile, Ac-KHPHLVRQKR-AMC would contribute as one informative component, but its reliability must be validated prospectively in larger, independent cohorts.

Follow-up analyses showed no clear correlation between protease activity and clinical phase (acute vs. remission), suggesting that protease dysregulation may be an inherent feature of IBD, independent of inflammatory severity. This aligns with recent findings of elevated serum furin levels in UC patients, regardless of disease activity^42^. These results challenge the assumption that increased protease activity reflects only acute inflammation and raise questions about the mechanisms sustaining this dysregulation. Notably, protease activity may persist even in the absence of symptoms, pointing to its potential as a pre-symptomatic biomarker. Further research is needed to evaluate its role in tracking disease progression or predicting flare-ups before clinical signs appear.

In conclusion, this study supports further investigation of protease profiling as a non-invasive approach that may help distinguish IBS from IBD and differentiate UC from CD. IBD samples showed diverse protease activity across multiple enzyme classes, while IBS was characterized by predominant serine protease—particularly furin-like—activity. These findings highlight the potential clinical relevance of furin-like enzymes and the need for further research into their role in gastrointestinal pathology. In our small follow-up subset, protease activity in IBD did not show a consistent correlation with disease phase, and some degree of dysregulation appeared to persist in remission. While this observation is preliminary, it is compatible with recent studies reporting protease alterations beyond overt inflammation and raises the hypothesis that protease profiling might contribute to earlier detection or risk stratification if validated in larger cohorts.

A key limitation of this study is the limited number of IBS patients, which restricts the statistical power of comparisons involving this group. Therefore, conclusions regarding IBS should be viewed as preliminary. Nevertheless, these early data provide valuable insights that can guide future research with larger, more balanced cohorts.

While larger studies are needed to validate these findings and enhance diagnostic precision, our results lay the groundwork for developing non-invasive tools based on enzymatic signatures. Expanding the substrate library may increase sensitivity and uncover additional biomarkers, advancing diagnostic capabilities in gastroenterology and reducing reliance on invasive procedures.

## Methods

### Human sample collection

A total of 42 participants (aged 15–81 years) were recruited from the Azienda Ospedale Università Padova, Italy, between April 2023 and May 2024. The cohort included 23 IBD patients (12 with CD and 11 with UC), 4 IBS patients, and 12 Healthy Controls (HC). It should be noted that the number of IBS patients included (*n* = 4) was limited due to the strict inclusion criteria and the difficulty in recruiting individuals willing to provide fecal samples. Consequently, the observations for this cohort should be regarded as preliminary and exploratory. Stool samples from IBD patients were collected during the acute phase of hospitalization, before initiation of any treatment known to alter protease activity (e.g., corticosteroids, antibiotics, biologics, or small-molecule therapies). Several patients were on ongoing outpatient maintenance therapy with mesalazine prior to admission. Additionally, 3 stool samples from the same patients (2 with CD and 1 with UC already included in the cohort) were collected during their remission phase and stored following the same protocol. Each patient IBD diagnosis was carried out according to ECCO guidelines^43^. Detailed clinical characteristics – including Montreal classification, disease duration, disease location/behaviour, biomarker indices (CRP, fecal calprotectin), and available endoscopic severity – are provided in Supplementary Table 1. During the stool collection and testing, patients were receiving pharmacological therapy with mesalazine under the supervision of their gastroenterologist. Patients with infectious diseases, including viral infections, were excluded from this study. All participants provided written informed consent before inclusion. The study was conducted in accordance with the ethical standards of the 1964 Declaration of Helsinki and its amendments and was approved by the Regional Ethical Committee for Clinical Trials (3312/AO/14).

### Sample extraction

For each stool sample collected, a small portion was homogenized by vortexing with MilliQ water at a ratio of 5 mL of water per 1 gram of stool^35^. After homogenization, the mixture was centrifuged at 4 °C at 5000 rpm for 15 min. Pellet was discarded, and supernatant filtered through a 0.45 μm PVDF filter. Extracts were stored at -20 °C and tested within one month of extraction.

### Substrate library design and synthesis

The peptide sequences used in this study were derived from key proteins involved in gastrointestinal integrity and inflammation, designed to assess proteolytic activity in IBD and IBS (Table 1). The interpretation of these substrates is substrate-centric rather than enzyme-centric: each peptide motif is known to be preferentially cleaved by a given class of proteases (e.g., trypsin-like, cathepsin-like, furin/PC-family), but we fully acknowledge that in complex biological matrices, multiple enzymes may contribute to their cleavage. Importantly, all substrates are AMC-based and release fluorescence only upon terminal peptide bond cleavage, which reduces the promiscuity typical of internally quenched substrates and enables a more robust discrimination of motif-specific susceptibility. Contactin-1 (CNTN-1) substrates (Ac-DGPYSLVA-AMC and Ac-GDGPYSLVA-AMC) correspond to residues 791–799 and 790–799, mimicking a crucial cell adhesion molecule that supports neuromuscular communication and immune homeostasis. Desmoglein-2 (DSG2) substrates (Ac-PHLVRQKR-AMC and Ac-KHPHLVRQKR-AMC) map to residues 42–49 and 39–49 of this desmosomal cadherin, essential for epithelial barrier function, which is compromised in IBD. Additional substrates (Ac-RRHL-AMC, Ac-RRPL-AMC, Ac-RSVL-AMC, Ac-RRLQ-AMC) were selected from proteins associated with cellular turnover and injury, key processes in inflammatory conditions. Ac-VFRSLK-AMC, derived from pleiotropic regulator 1 (PLRG1, residues 14–19) and Subtilisin-kexin-isozyme-1 (SKI-1) / Site-1 protease (S1P) (residue 142–147), links to immune regulation and cholesterol metabolism, while Ac-ERSLK-AMC, from Glucosidase II beta subunit (GIIβ, residues 381–385) and Maltase-glucoamylase (MGAM, residues 1409–1413), is implicated in glycoprotein folding and starch digestion, relevant to IBS and IBD. Ac-PANQRRHL-AMC and Ac-ANQRRHLL-AMC, corresponding to ATF6 (residues 411–418 and 412–419), are linked to intestinal inflammation and stress responses. Positive controls included Pyr-RTKR-AMC (furin/trypsin substrate) and R-AMC (trypsin/cathepsin substrate), as these proteases are known to be upregulated in gastrointestinal pathologies. Each sequence was selected based on its accessibility to enzymatic cleavage, ensuring its relevance in disease-associated proteolytic activity (Fig. S6). Contactin-1 and Desmoglein-2 substrates were synthesized in-house, using Fmoc-based solid-phase synthesis, purified by HPLC, and confirmed by MALDI-TOF. Additional substrates, including PLRG1-, GIIβ-, MGAM-, and ATF6-derived peptides, were custom-synthesized by GenScript (generous gift from Dr. Sylvia Rothenberger). Standard fluorogenic controls (Pyr-RTKR-AMC and R-AMC) were purchased from Peptide Institute, Inc. and Bachem AG.

### Intrinsic fluorescence assessment

Before substrate addition, each stool extract was measured under assay conditions (extract + buffer only; Ex 365 nm / Em 460 nm) to quantify intrinsic fluorescence. In samples with high baseline signal, an additional high-speed centrifugation step was performed to remove particulates; however, intrinsic fluorescence remained unchanged, indicating that the signal originated from soluble fluorophores rather than scattering (Fig. S1). Extracts showing excessively high intrinsic fluorescence were excluded from enzymatic analyses. For all other samples, intrinsic background was recorded and used to support interpretation of kinetic traces.

### Fluorescence enzymatic assay

In vitro assays were carried out in flat black 96-well plates. Measurements were taken using an Infinite^®^ 200 Pro Tecan plate reader with a kinetic cycle of 1 h and 15 min at room temperature (RT), with one measurement every 5 min. The settings for fluorescent readings were as follows: excitation wavelength at 365 nm, emission wavelength at 460 nm. For each enzymatic digestion, a final concentration of 5 µM fluorescent AMC substrates was used. The reaction mixture included 20 µL of stool extract diluted 1:20 in MilliQ water, bringing the total volume to 100 µL with additional MilliQ water. The digestion was carried out in the appropriate buffer (2 mM CaCl_2_ and 25 mM Sodium Acetate at pH 5.5; 2 mM CaCl_2_ and 25 mM 4-(2-hydroxyethyl)-1-piperazineethanesulfonic acid (HEPES) at pH 6.5; and 2 mM CaCl_2_ and 25 mM tris(hydroxymethyl)aminomethane (Tris-HCl) at pH 7.5 and pH 8.0).

### Assay calibration, controls, and data extraction

To ensure that fluorescence intensity reflected enzymatic reaction velocity under our experimental conditions, several controls and analytical steps were implemented. For each kinetic digestion, reaction rates were calculated exclusively from the initial linear portion of the fluorescence trace (typically the first 5–20 min), where AMC release is known to be proportional to proteolytic activity. This prevents nonlinear effects related to substrate depletion or product accumulation.

Each plate included substrate-only wells, plate-background wells, and intrinsic fluorescence wells (containing each stool extract without substrate). Because autofluorescence varies between stool samples, the intrinsic fluorescence trace of each extract was subtracted from its corresponding digestion curve before slope calculation. All reactions were performed in independent triplicates, and plate-to-plate variability was minimized using a normalization factor derived from reference background wells.

Peptide substrates used in this study had been previously validated in related biochemical work^44^; DSG-derived peptides were cleaved by recombinant furin (Fig. S3); in those studies, furin batch activity was verified using the RVLK-AMC substrate.

Importantly, because stool extracts contain complex mixtures of host- and microbiota-derived proteases, endogenous inhibitors, mucins, and variable ionic environments, calibration curves based on single purified enzymes cannot reproduce the composite enzymatic activity present in stool. For this reason, the assay was designed to derive relative proteolytic signatures across substrates and pH conditions rather than to quantify absolute concentrations of individual enzymes.

### Inhibitor test with furin inhibitor decanoyl-RVKR chlorometylketone

Each enzymatic digestion was prepared by combining 20 µL of stool extract, previously diluted 1:20 in MilliQ water, with a buffer containing a final concentration of 2 mM CaCl_2_ and 25 mM Tris-HCl at pH 7.5. The mixture was then brought up to a final volume of 100 µL with additional MilliQ water. Before adding the substrate (5 µM Pyr-RTKR-AMC), 2 µM of the Furin inhibitor Decanoyl-RVKR chlorometylketone was introduced into each well to neutralize potential furin-like proteases. As controls, the same samples were tested with 2 µM of DMSO in triplicate. In vitro assays were conducted in flat black 96-well plates as described above.

### Thin-layer chromatography (TLC)

5 µL of stool extracts, diluted 1:20 in MilliQ water, were spotted onto a thin layer silica plate. Alongside this, 5 µL of an aqueous solution of two different commercially available drug containing Mesalazine at 1 mg/mL were spotted. After drying, the very bottom edge of the TLC plate was immersed in a solution of ethyl acetate: butanol: acetic acid: milliQ water = 5:3:1:1, v/v. The solvent was allowed to ascend the plate by capillarity for 30 min. Following solvent removal, the TLC plate was exposed to ultraviolet light at 405 nm to detect any likely fluorescent components.

### Statistical considerations

All enzymatic assays were conducted in triplicate to ensure reproducibility and reliability of the results. Fluorescence activity was monitored at 5-minute intervals over a 75-minute period during each kinetic cycle. To account for any potential background interference, the fluorescence readings for each plate were normalized. This was achieved by measuring the background fluorescence from both the extract (prepared as a 20% solution in MilliQ water) and the empty wells on the plate. The normalization factor for each plate was calculated as follows:

$$\:Normalization\:factor\:\left(plate\:x\right)=\frac{mean\:emply\:well\:plate\:x}{reference\:value}$$

where the reference value represents the mean fluorescence of empty wells from a designated reference plate. The data obtained from each plate were then adjusted by dividing by the respective normalization factor, ensuring consistency across all experimental conditions.

Kinetic data were expressed as relative fluorescence units (RFU) per minute, with mean and standard deviation calculated from triplicates across pH levels and conditions. Enzymatic activity was quantified by measuring the slope of the linear phase of each curve, where substrate degradation was most active, using GraphPad Prism. These slopes were plotted by pH and substrate to compare activity across HC, CD, UC, and IBS groups. For follow-up IBD cases, initial and remission samples were also compared to healthy controls.

### Statistical analysis

To determine the statistical significance of differences observed between the IBD, IBS, and HC groups, unpaired t-tests were conducted using a 95% confidence interval using GraphPad prism software. This statistical analysis, which assumes normally distributed data with equal standard deviations, was essential for validating the observed variations in enzymatic activity between the groups under study.

## Supplementary Information

Below is the link to the electronic supplementary material.

Supplementary Material 1

Supplementary Material 2

Supplementary Material 3

Supplementary Material 4

Supplementary Material 5

Supplementary Material 6

Supplementary Material 7

Supplementary Material 8

Supplementary Material 9

## Data Availability

The data, analytic methods, and study materials used in this study will be made available to other researchers upon reasonable request. Researchers interested in accessing the data can contact the corresponding authors, Dr. Antonella Pasquato (antonella.pasquato@unipd.it) or Dr. Edoardo Vincenzo Savarino (edoardo.savarino@unipd.it). Any shared data will comply with ethical and confidentiality guidelines.
