# Supplementary figures and images for: Protease profiling in fecal samples: a novel non-invasive diagnostic tool for gastrointestinal disorders
Source: Sci Rep. 2025 Dec 17;16:2444. doi: 10.1038/s41598-025-32301-6 (PMC12820393; doi:10.1038/s41598-025-32301-6)

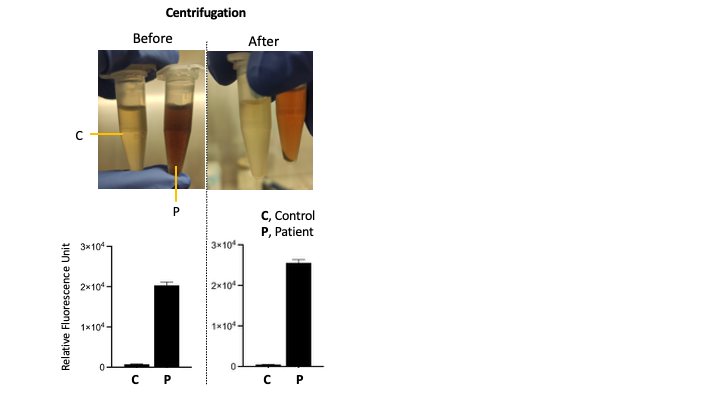

Supplement: Supplementary file 1 — Supplementary Material 1 [file 41598_2025_32301_MOESM1_ESM.tiff]

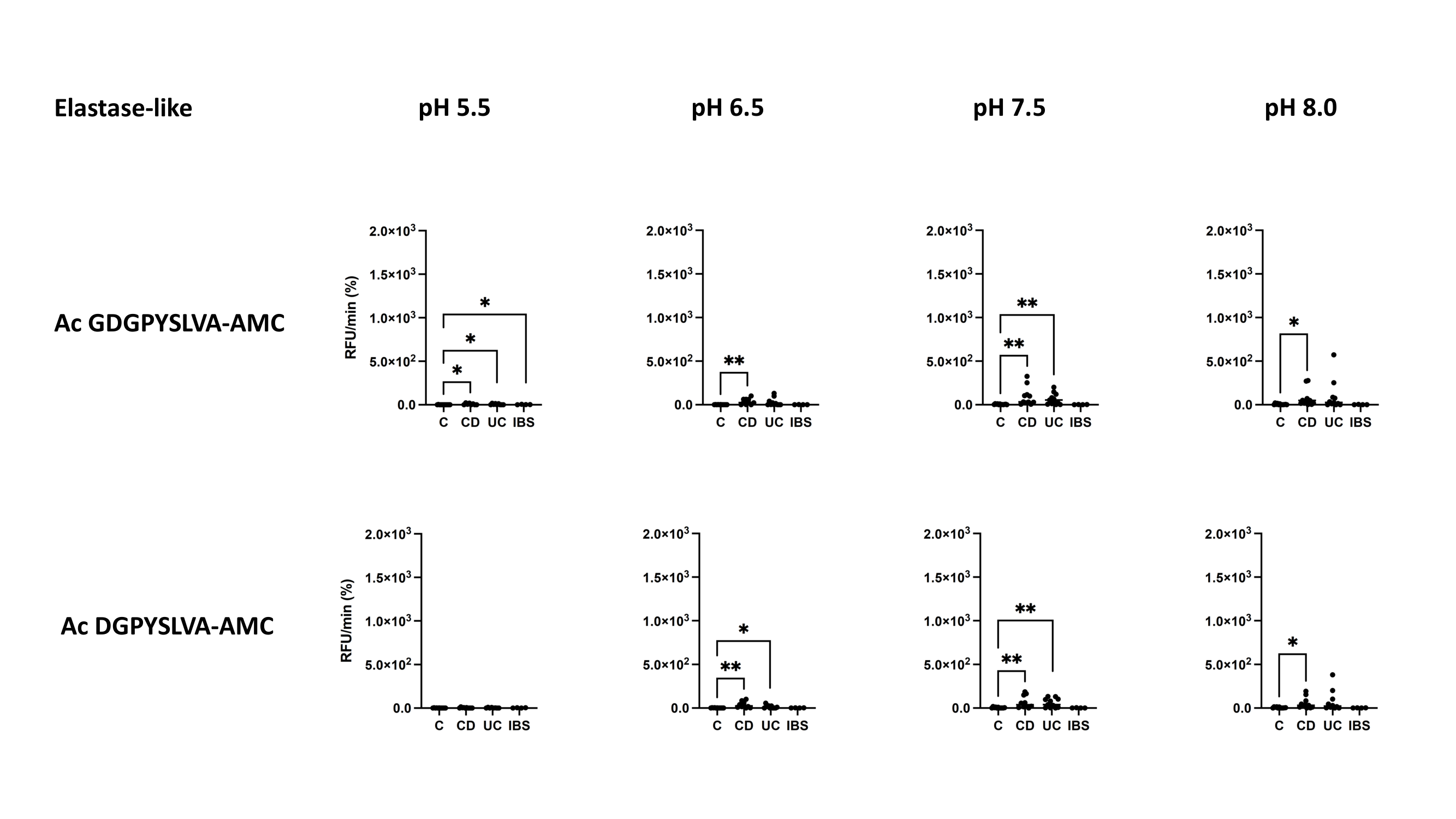

Supplement: Supplementary file 2 — Supplementary Material 2 [file 41598_2025_32301_MOESM2_ESM.tiff]

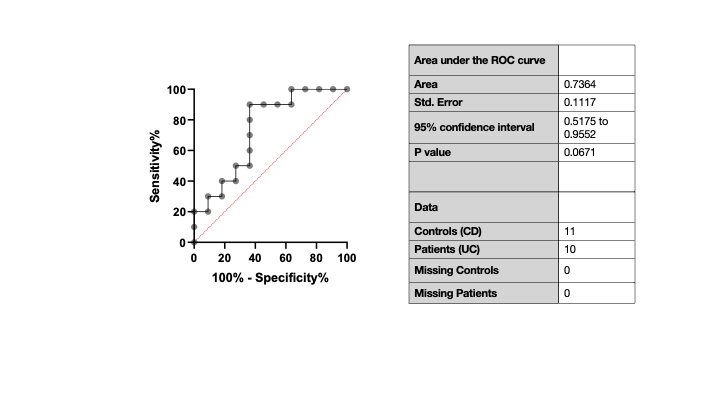

Supplement: Supplementary file 3 — Supplementary Material 3 [file 41598_2025_32301_MOESM3_ESM.tiff]

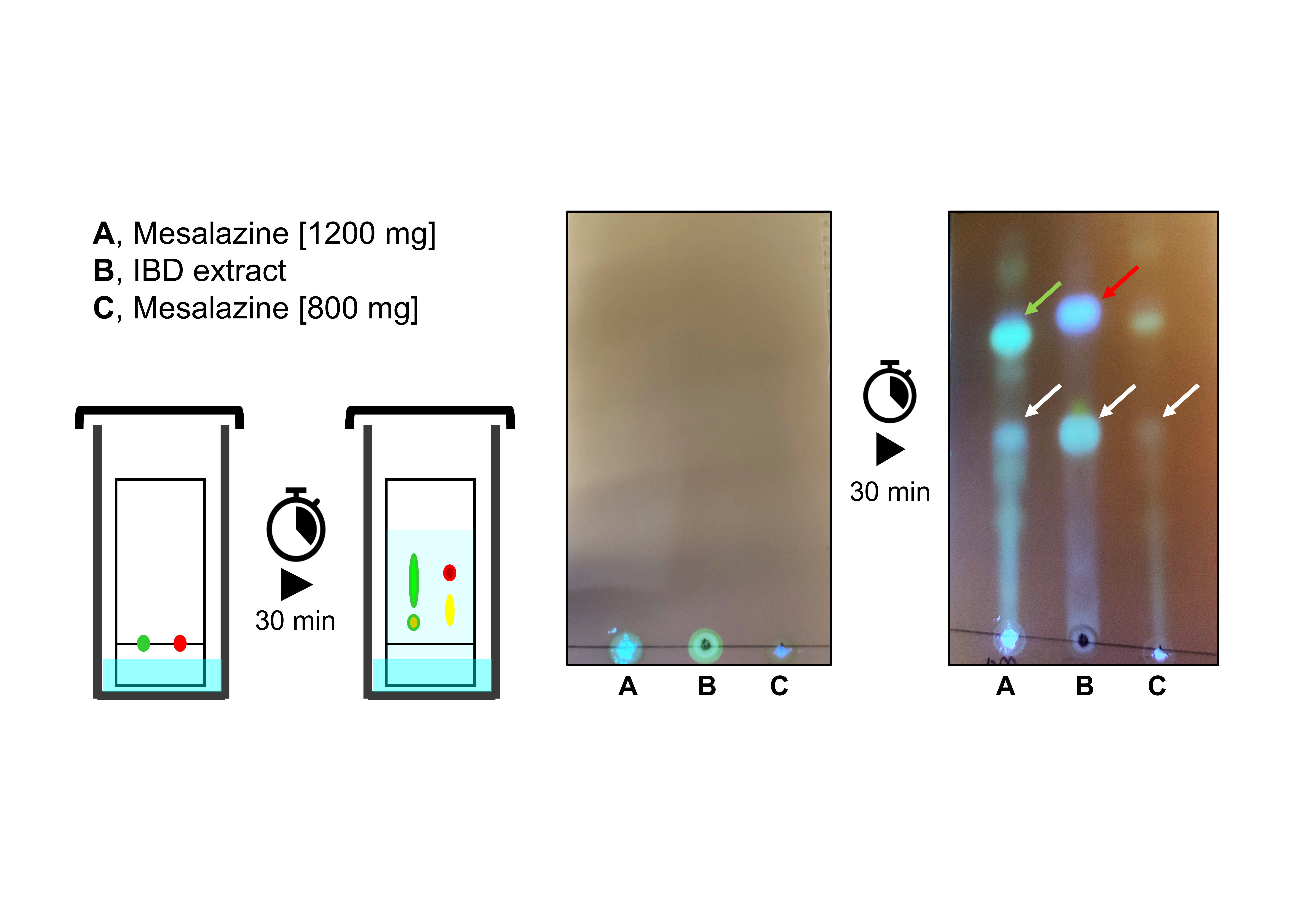

Supplement: Supplementary file 7 — Supplementary Material 7 [file 41598_2025_32301_MOESM7_ESM.tiff]

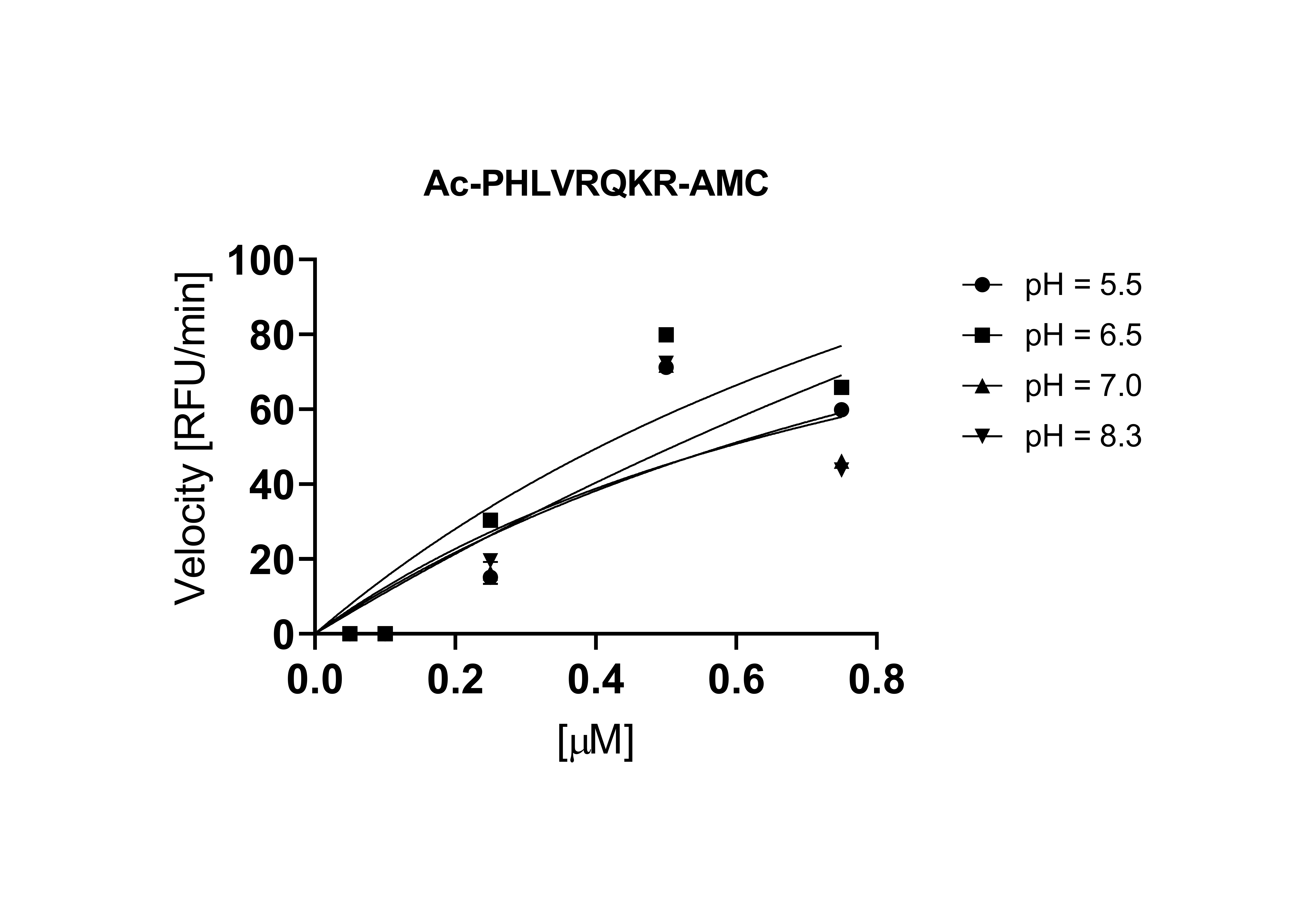

Supplement: Supplementary file 8 — Supplementary Material 8 [file 41598_2025_32301_MOESM8_ESM.tiff]

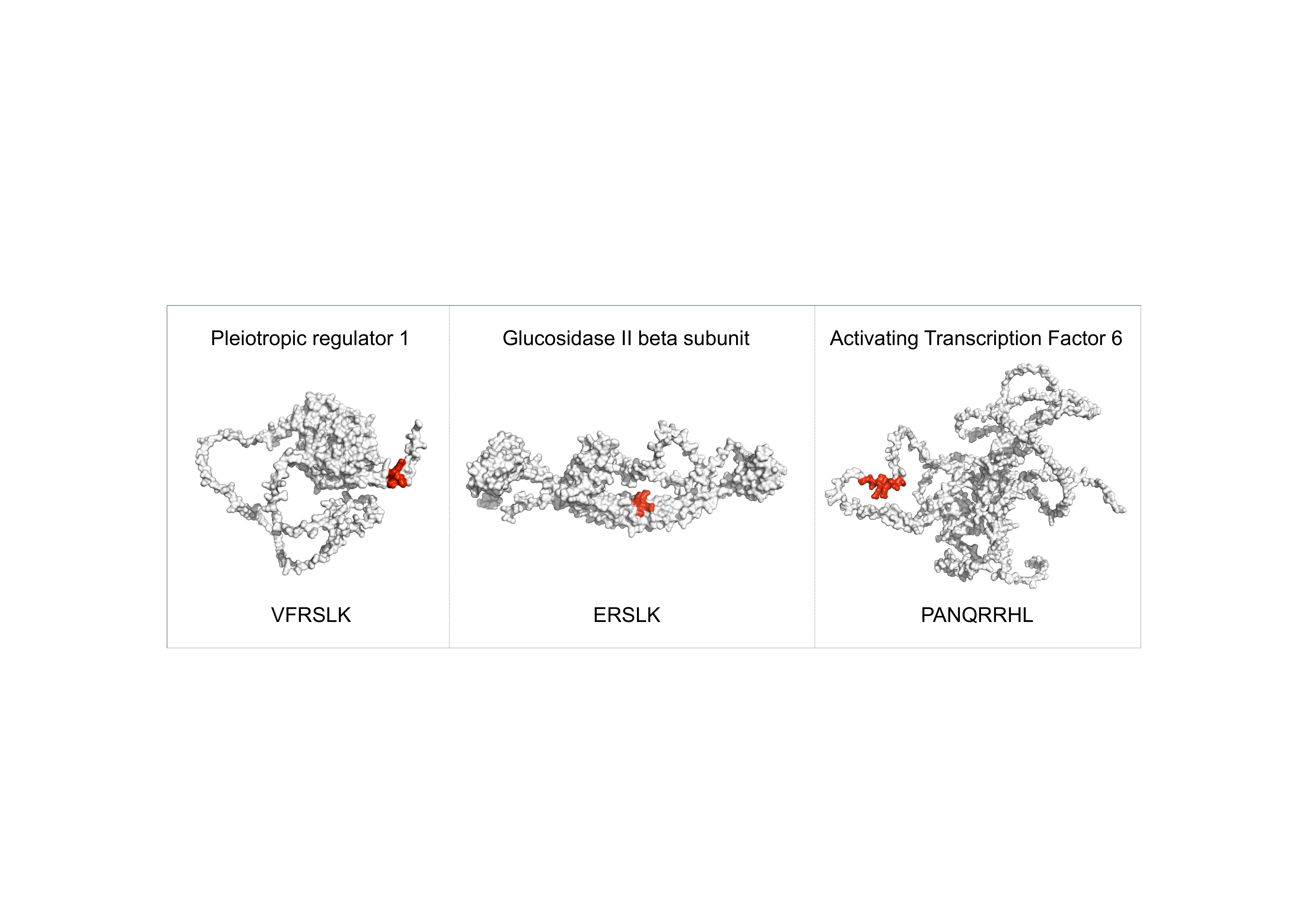

Supplement: Supplementary file 9 — Supplementary Material 9 [file 41598_2025_32301_MOESM9_ESM.tiff]
