# Supplementary material for: Protease profiling in fecal samples: a novel non-invasive diagnostic tool for gastrointestinal disorders
Source: Sci Rep. 2025 Dec 17;16:2444. doi: 10.1038/s41598-025-32301-6 (PMC12820393; doi:10.1038/s41598-025-32301-6)
Supplement: Supplementary file 4 — Supplementary Material 4 [file 41598_2025_32301_MOESM4_ESM.docx]

| **Class** | **Enzyme** | **Disease** | **Reference** |
| --- | --- | --- | --- |
| **Matrix Metalloproteinases (MMPs)** | MMP-1 | IBD | [1] |
|  | MMP-13 | IBD | [2,3] |
|  | MMP-2 | UC | [4] |
|  | MMP-2 | CD | [5] |
|  | MMP-7 | IBD | [3] |
|  | MMP-7 | UC | [4] |
|  | MMP-9 | IBD | [6,7] |
|  | MMP-7 | UC | [4] |
| **Serine Proteinases** | Tryptase | IBS | [8] |
|  | Tryptase | UC | [9] |
|  | Trypsin | IBS | [8] |
|  | Trypsin | IBD | [10,11] |
|  | Calpain-8 | IBS | [12] |
|  | Elastase 2A | IBD | [13] |
|  | Neutrophil Elastase | IBD | [14] |
|  | Cathepsin G | IBD | [10] |
| **Aspartic** | Cathepsin D | IBD | [15] |
| **Cysteine** | Cathepsin B and L | IBD | [16] |

**Table S1.** **Overview of Protease Classes and Their Associations with Gastrointestinal Disorders** This table summarizes key protease classes, including matrix metalloproteinases (MMPs), serine proteases, cysteine proteases, that are implicated in inflammatory bowel disease (IBD), Crohn’s disease (CD), ulcerative colitis (UC), and irritable bowel syndrome (IBS).

**REFERENCES**

1. De Bruyn, M. *et al.* Infliximab Restores the Dysfunctional Matrix Remodeling Protein and Growth Factor Gene Expression in Patients with Inflammatory Bowel Disease: *Inflammatory Bowel Diseases* **20**, 339–352 (2014).

2. Vizoso, F. J. *et al.* Collagenase-3 (MMP-13) expression by inflamed mucosa in inflammatory bowel disease. *Scandinavian Journal of Gastroenterology* **41**, 1050–1055 (2006).

3. Rath, T. *et al.* Enhanced expression of MMP-7 and MMP-13 in inflammatory bowel disease: A precancerous potential? *Inflammatory Bowel Diseases* **12**, 1025–1035 (2006).

4. Jakubowska, K. *et al.* Expressions of Matrix Metalloproteinases (MMP-2, MMP-7, and MMP-9) and Their Inhibitors (TIMP-1, TIMP-2) in Inflammatory Bowel Diseases. *Gastroenterol Res Pract* **2016**, 2456179 (2016).

5. Wang, D. *et al.* Fibroblast-Mediated MMP2 Contribution to Nonresponse in Anti-TNFα Therapy for Crohn’s Disease. *Inflammatory Bowel Diseases* izaf263 (2025) doi:10.1093/ibd/izaf263.

6. Tarlton, J. F. *et al.* The Role of Up-Regulated Serine Proteases and Matrix Metalloproteinases in the Pathogenesis of a Murine Model of Colitis. *The American Journal of Pathology* **157**, 1927–1935 (2000).

7. Baugh, M. D. *et al.* Matrix metalloproteinase levels are elevated in inflammatory bowel disease. *Gastroenterology* **117**, 814–822 (1999).

8. Cenac, N. *et al.* Role for protease activity in visceral pain in irritable bowel syndrome. *J Clin Invest* **117**, 636–647 (2007).

9. Hamilton, M. J. *et al.* Essential role for mast cell tryptase in acute experimental colitis. *Proc Natl Acad Sci U S A* **108**, 290–295 (2011).

10. Jablaoui, A. *et al.* Fecal Serine Protease Profiling in Inflammatory Bowel Diseases. *Frontiers in Cellular and Infection Microbiology* **10**, (2020).

11. Denadai-Souza, A. *et al.* Functional Proteomic Profiling of Secreted Serine Proteases in Health and Inflammatory Bowel Disease. *Scientific Reports* **8**, 7834 (2018).

12. Swan, C. *et al.* Identifying and testing candidate genetic polymorphisms in the irritable bowel syndrome (IBS): association with TNFSF15 and TNFα. *Gut* **62**, 985–994 (2013).

13. Motta, J.-P. *et al.* Epithelial production of elastase is increased in inflammatory bowel disease and causes mucosal inflammation. *Mucosal Immunology* **14**, 667–678 (2021).

14. Górecka, A. & Komosinska-Vassev, K. Neutrophil Elastase and Elafin in Inflammatory Bowel Diseases: Urinary Biomarkers Reflecting Intestinal Barrier Dysfunction and Proteolytic Activity. *JCM* **14**, 2466 (2025).

15. Hausmann, M. *et al.* Cathepsin D is up-regulated in inflammatory bowel disease macrophages. *Clinical and Experimental Immunology* **136**, 157–167 (2004).

16. Menzel, K. *et al.* Cathepsins B, L and D in inflammatory bowel disease macrophages and potential therapeutic effects of cathepsin inhibition *in vivo*. *Clinical and Experimental Immunology* **146**, 169–180 (2006).
