# Supplementary material for: Protease profiling in fecal samples: a novel non-invasive diagnostic tool for gastrointestinal disorders
Source: Sci Rep. 2025 Dec 17;16:2444. doi: 10.1038/s41598-025-32301-6 (PMC12820393; doi:10.1038/s41598-025-32301-6)
Supplement: Supplementary file 5 — Supplementary Material 5 [file 41598_2025_32301_MOESM5_ESM.docx]

| **Patient ID** | **Diagnosis** | **Sex** | **Age** | **Year of first diagnosis** | **Montreal Location (CD)** | **Montreal Behaviour (CD)** | **Montreal Extension (UC)** | **Comorbidity** | **Surgery** | **Fecal calprotectin [µg/g]** | **CRP [mg/L]** |
| --- | --- | --- | --- | --- | --- | --- | --- | --- | --- | --- | --- |
| P01 | UC | M | 72 | 2014 | N/A | N/A | E2 | Knee arthralgia, fatigue, and memory impairment | None | 1768,0 | 51,68 |
| P02 | CD | F | 59 | 2016 | L2 | B1 | N/A | None | None | 1633,0 | 9,36 |
| P03 | UC | F | 69 | 2017 | N/A | N/A | E2 | Spondyloarthritis, osteoporosis | None | 149,6 | 4,86 |
| P04 | CD (initially UC) | M | 58 | 2023 | L2 | B3p | N/A | History of erythema nodosum and oral aphthae | Drainage of perianal abscess and seton placement (2023). | 643,0 | 47,73 |
| P05 | CD | F | 50 | 2020 | L3 | B2 | N/A | Joint pain in the lower limbs and lumbar region | Extended right hemicolectomy including transverse colon for double colonic stricture (2020); Ileo-colic resection for subocclusion (2021), complicated by intra-abdominal collection requiring drainage (July 2021); Pneumatic dilation of ileo-colic anastomosis (2023) | 895,4 | 5,14 |
| P06 | UC | F | 46 | 1998 | N/A | N/A | E3 | Psoriasis, oral pemphigus | None | 140,0 | 0,83 |
| P07 | UC | M | 66 | 2000 | N/A | N/A | E3 | None | None | 1183,0 | 61,98 |
| P08 | CD | M | 68 | 2020 | L1 | B2 | N/A | None | None | 252,8 | 10,70 |
| P09 | UC | M | 15 | 2022 | N/A | N/A | E3 | None | None | 4737,0 | 15,82 |
| P10 | UC | F | 39 | 2014 | N/A | N/A | E2 | Erythema nodosum in 2021 | None | 952,4 | 42,55 |
| P11 | CD | F | 65 | 2018 | L1 | B1 | N/A | None | None | 187,3 | 77,82 |
| P12 | CD | F | 81 | 2023 | L2 | B3p | N/A | Pyoderma gangrenosum on peristomal skin | Previous hystero-annexectomy and appendectomy; Lateral colostomy (2023). | N/A | 1,87 |
| P13 | CD | M | 47 | 1996 | L1 | B2 | N/A | None | Ileal resection (2007) | 20,5 | 2,89 |
| P14 | CD | M | 30 | 2011 | L1 | B2 | N/A | None | None | 268,8 | 4,76 |
| P15 | UC | M | 63 | 2023 | N/A | N/A | E3 | None | None | N/A | 1,16 |
| P16 | UC | F | 47 | 2011 | N/A | N/A | E2 | None | None | N/A | 12,75 |
| P17 | CD | M | 43 | 1988 | L3 | B3p | N/A | Erythematous plaques and fistulous tracts in the perianal region | Ileocolic resection with ileo-ascending anastomosis and sigmoid repair (2009); Extended right hemicolectomy and resection of intestinal loops involved by multiple fistulous tracts (2017). | N/A | 6,17 |
| P18 | UC | F | 49 | 2018 | N/A | N/A | E2 | None | None | 676,0 | 20,48 |
| P19 | UC | F | 21 | 2024 | N/A | N/A | E2/3 | None | None | 1743,0 | 3,55 |
| P20 | UC | F | 69 | N/A | L3 | B3p | NA | N/A | N/A | 149,6 | 4,86 |
| P21 | CD | M | 20 | N/A | NA | NA | E2/3 | N/A | N/A | 468,1 | 8,19 |
| P22 | UC | F | 75 | N/A | NA | NA | E3 | N/A | N/A | 3706,0 | 14,33 |
| P23 | CD | F | 24 | N/A | L3 | B3p | NA | N/A | N/A | 1578,0 | 56,54 |

**Table S2**. Clinical and demographic features of IBD patients included in the study, including age, sex, diagnosis (UC, CD, IBS), year of first diagnosis, disease location, surgical history, comorbidities, and available inflammatory markers (CRP and fecal calprotectin). All information was obtained from medical records at the time of stool collection, and the reported CRP value corresponds to the measurement performed at the time of sample acquisition.

N/A indicates not available, meaning that the information was either not recorded in the clinical documentation or not applicable to the patient’s condition. CRP = C-reactive protein.
