# Supplementary material for: Protease profiling in fecal samples: a novel non-invasive diagnostic tool for gastrointestinal disorders
Source: Sci Rep. 2025 Dec 17;16:2444. doi: 10.1038/s41598-025-32301-6 (PMC12820393; doi:10.1038/s41598-025-32301-6)
Supplement: Supplementary file 6 — Supplementary Material 6 [file 41598_2025_32301_MOESM6_ESM.docx]

**Figure S1.** **Assessment of intrinsic fluorescence and contribution of particulates in stool extracts.** Representative stool extracts from a healthy control (C) and a patient sample (P) were imaged before and after an additional high-speed centrifugation step to remove residual particulates. Although centrifugation clarified the supernatant, the patient extract retained a markedly stronger intrinsic coloration.

Intrinsic fluorescence (Ex 365 nm / Em 460 nm) was measured in the same extracts incubated with buffer only (no substrate). The patient sample (P) displayed a strong, substrate-independent fluorescence signal both before and after clarification, whereas the control sample (C) showed minimal intrinsic fluorescence. These results indicate that the elevated background signal originates from soluble fluorescent compounds rather than from light scattering by particulates. Samples showing extremely high intrinsic fluorescence, such as sample P, were excluded from downstream kinetic analyses to avoid artefacts.


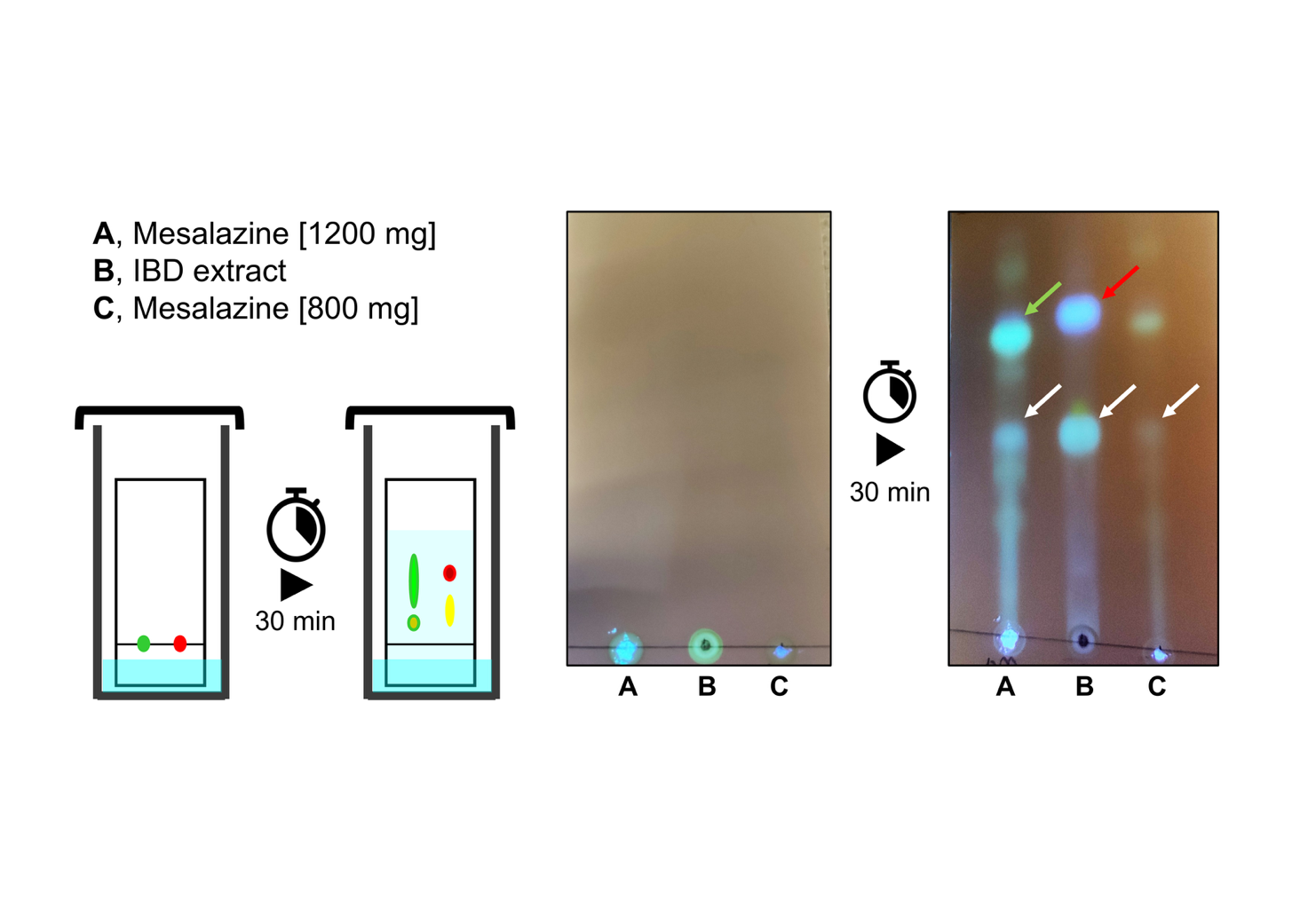


**Figure S2.** **Thin-Layer Chromatography (TLC) analysis of stool extract and mesalazine.** TLC was performed to identify the presence of mesalazine and its metabolites in stool samples (B) alongside mesalazine standards at concentrations of 1200 mg (A) and 800 mg (C). Samples were developed using a solvent system consisting of ethyl acetate, butanol, acetic acid, and milli-Q water in a 5:3:1:1 v/v ratio, for 30 minutes. Fluorescent spots were visualized under a UV lamp at 405 nm. The green arrow indicates the mesalazine component, and the red arrow points to a probable metabolite of mesalazine. White arrows mark additional fluorescent spots, present in all three lanes, suggesting the presence of undigested mesalazine or its derivatives in the stool sample, contributing to intrinsic fluorescence in the extract.


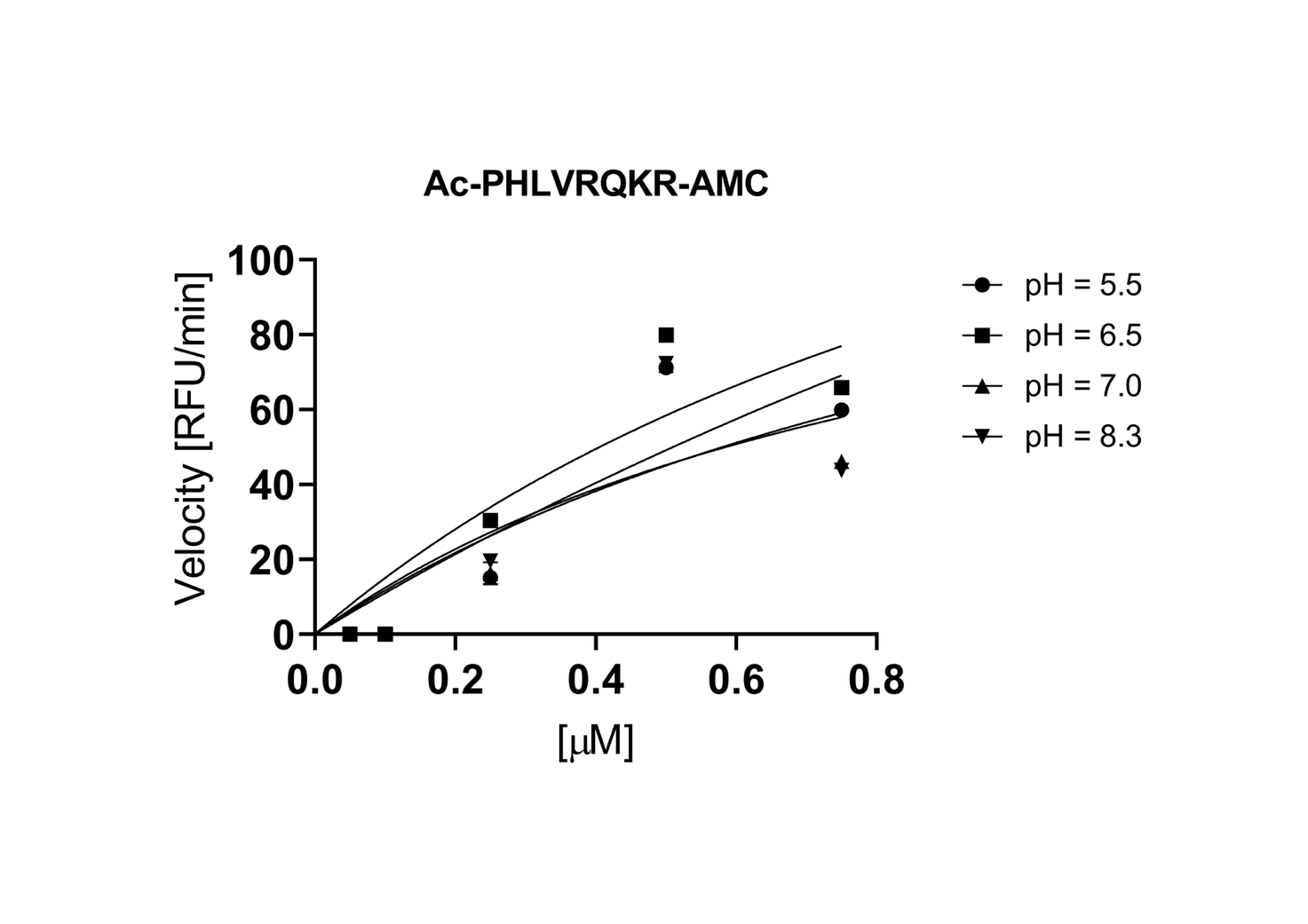


**Figure S3** Increasing concentrations of Ac-PHLVRQKR-AMC were incubated with soluble Furin across varying pH conditions. Fluorescence (excitation at 360 nm, emission at 460 nm) was monitored for 1 hour, and the reaction velocity was determined from the linear portion of the kinetic curve. Data represent the average of three independent measurements, with normalized velocity (RFU/min) plotted against substrate concentration. All analyses were performed using GraphPad Prism software, ensuring rigorous statistical validation of the enzymatic activity of Furin on the Desmoglein-2-derived peptide.


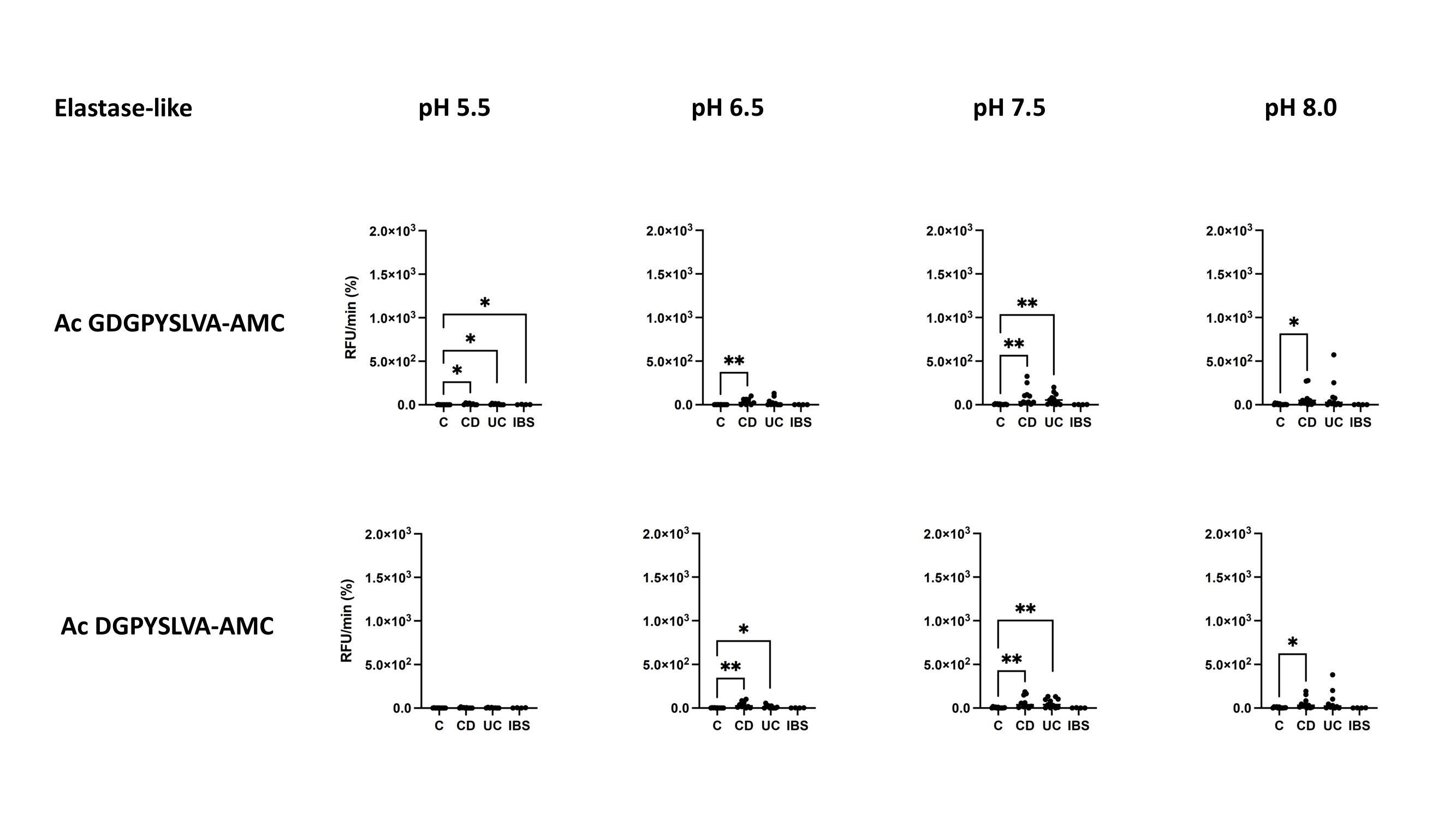


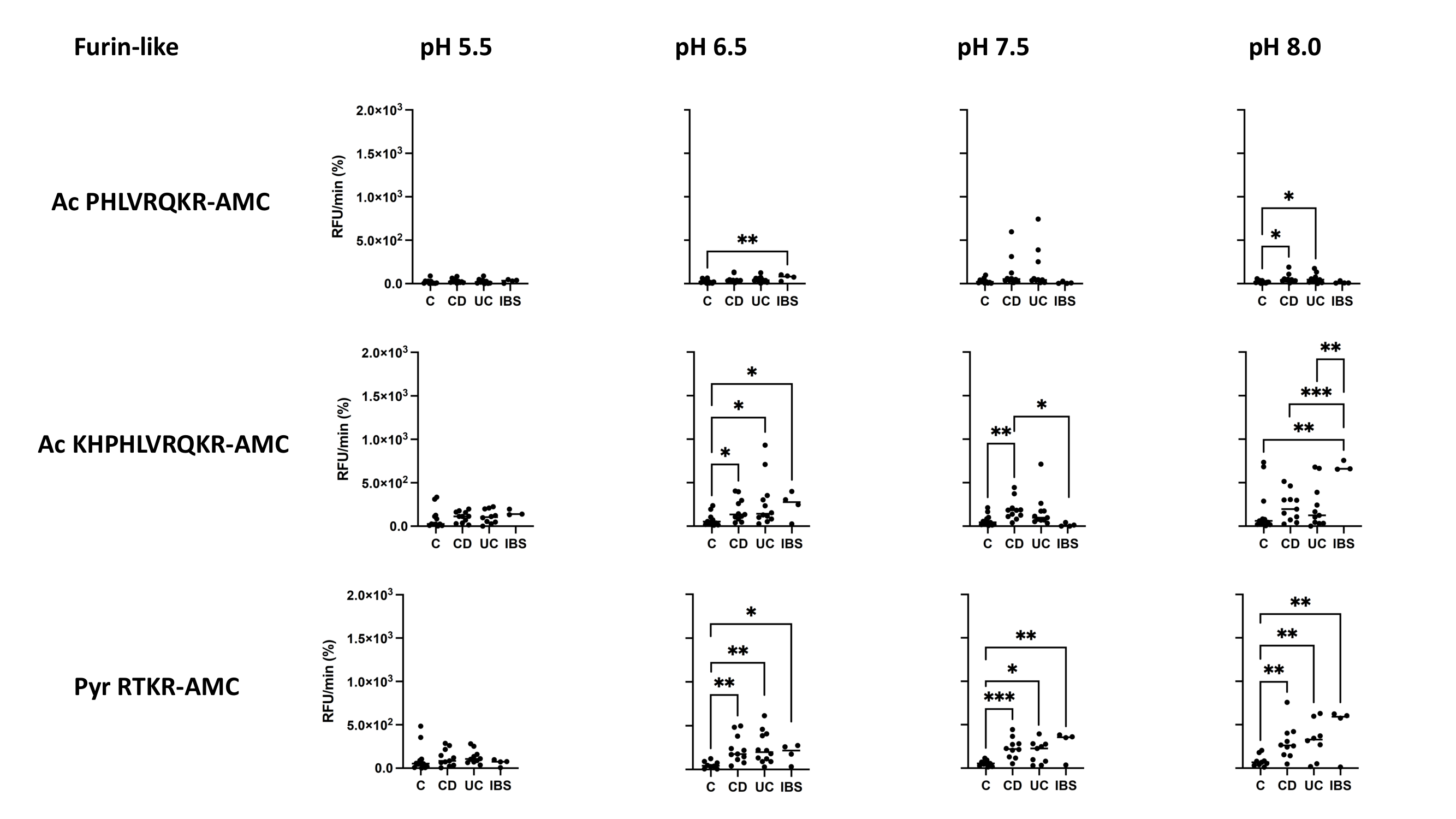


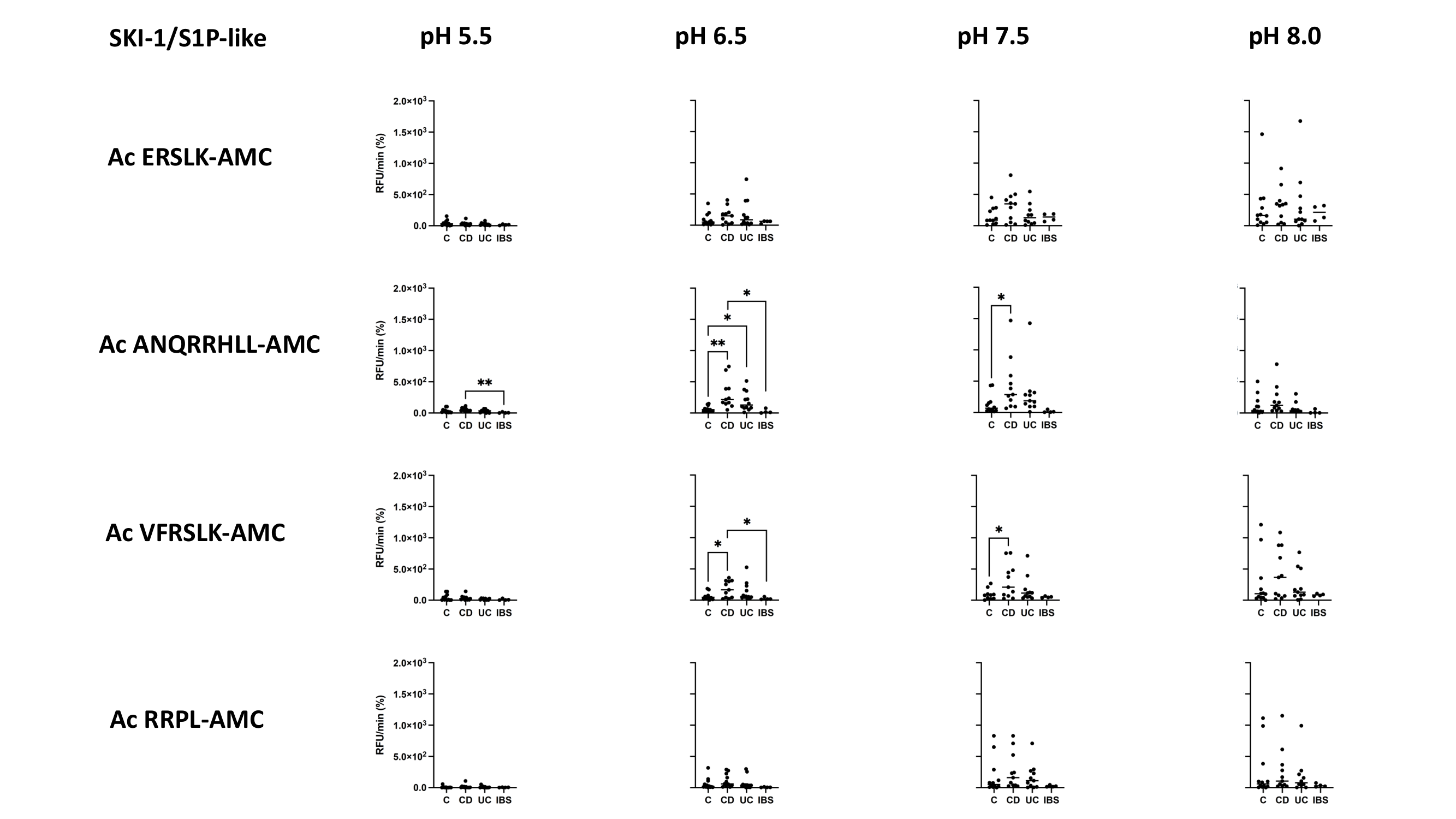

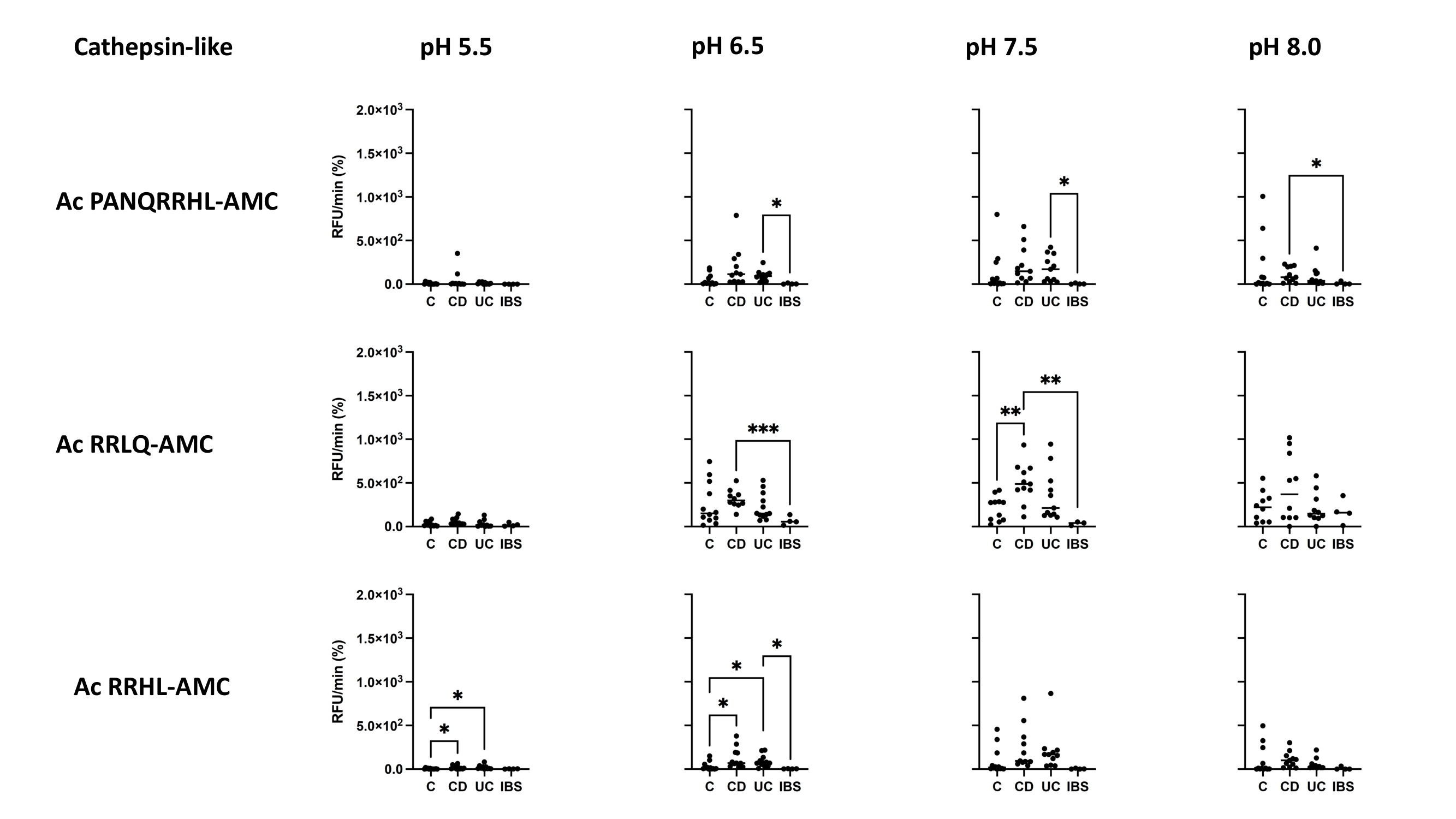

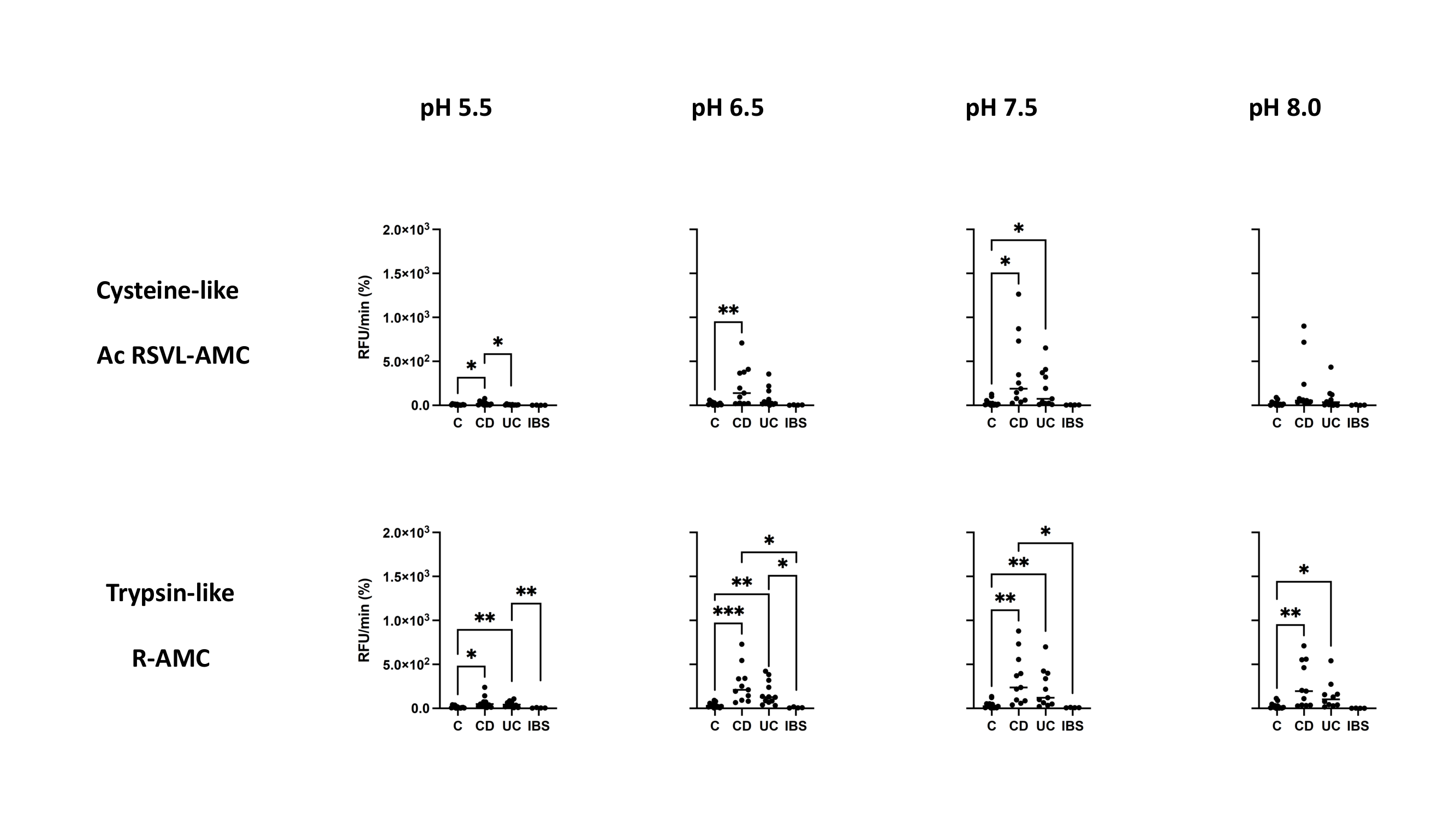


**Figure S4. IBD and IBS enzymatic assays results at pH 5.5, pH 6.5, pH 7.5, and pH 6.5** Protease activity profiles at pH 6.5 across different patient cohorts (Healthy Controls (C), CD, UC, and IBS). Enzymatic activity is displayed as Relative Fluorescence Units per minute (RFU/min) for 14 peptide substrates. Each dot represents the mean activity of triplicate measurements for an individual patient sample, with bars representing mean activity levels for each group. For the IBS group at pH 8 and Ac KHPHLVRQKR-AMC, three data points are displayed due to the exclusion of one dataset that did not meet quality criteria. Enzymatic assays were performed using substrates at 5 µM, with stool samples diluted 1:20 v/v in milli-Q water. Fluorescence measurements were collected every 5 minutes over 75 minutes. Kinetic data were normalized, and the linear portion of the curve was used to calculate RFU/min. Statistical significance between groups was evaluated using an unpaired t-test, with *p < 0.05, **p < 0.01, and ***p < 0.001.

**Figure S5 Receiver Operating Characteristic (ROC) analysis of Ac-RSVL-AMC activity at pH 5.5 for distinguishing ulcerative colitis (UC) from Crohn’s disease (CD)**.

The ROC curve was generated using individual activity values from UC (n = 10) and CD (n = 11) patients. The area under the curve (AUC) was 0.7364 (95% CI: 0.5175–0.9552; SE = 0.1117; p = 0.0671), indicating moderate discriminatory ability in this exploratory cohort. Given the limited sample size, these results should be interpreted as preliminary and hypothesis-generating, and larger independent cohorts will be required to assess diagnostic performance. Sensitivity and (100 – specificity) are plotted on the y- and x-axes, respectively.


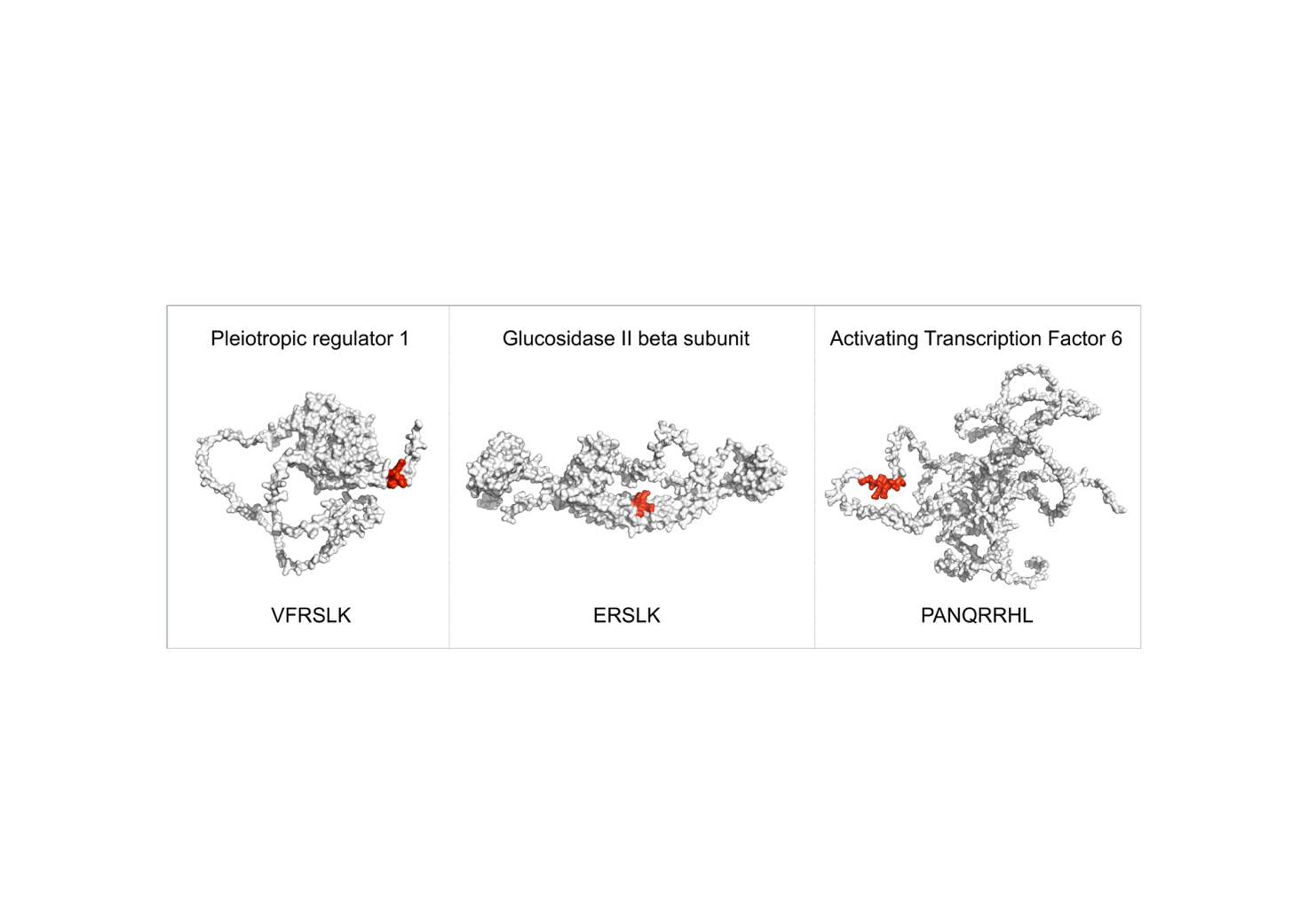


**Figure. S6 Peptide sequences are located in protein regions accessible to proteases.** The 3-D structures of Pleiotropic Regulator 1 (AF-O43660-F1), Glucosidase II Beta Subunit (AF-P14314-F1), and Activating Transcription Factor 6 (AF-P18850-F1) were obtained from the AlphaFold database. The relevant protein sequences of interest were highlighted in red using PyMOL to indicate the regions accessible for proteolytic cleavage.
